# Comprehensive Analysis of Clinical Significance, Immune Infiltration and Biological Role of m^6^A Regulators in Early-Stage Lung Adenocarcinoma

**DOI:** 10.3389/fimmu.2021.698236

**Published:** 2021-09-28

**Authors:** Bolun Zhou, Shugeng Gao

**Affiliations:** Department of Thoracic Surgery, National Cancer Center/National Clinical Research Center for Cancer/Cancer Hospital, Chinese Academy of Medical Sciences and Peking Union Medical College, Beijing, China

**Keywords:** m^6^A (N6-methyladenosine), lung adenocarcinoma, tumor microenvironment, immune infiltration, immunotherapy

## Abstract

Recent publications have revealed that N6-methyladenosine (m^6^A) modification is critically involved in tumorigenesis and metastasis. However, the correlation of m^6^A modification and immune infiltration in early-stage lung adenocarcinoma (LUAD) is still uncertain. We performed NMF clustering based on 23 m^6^A regulators and identify three distinct m^6^A clusters and three m^6^A related genes clusters (m^6^A cluster-R) in early-stage LUAD. The immune infiltrating levels were calculated using CIBERSORT, MCPcounter and ssGSEA algorithms. And we established the m^6^A-predictive score to quantify m^6^A modified phenotypes and predict immunotherapeutic responses. Based on the TME characteristics, different immune profiles were also identified among three m^6^A gene-related clusters. And the m^6^A-R-C2 was related to a favorable overall survival (OS), whereas m^6^A-R-C3 had unfavorable overall survival. The m^6^A-predictive score was built according to the expression levels of m^6^A-related genes, and patients could be stratified into subgroups with low/high scores. Patients with high scores had poor overall survival, enhanced immune infiltration, high tumor mutation burden and increased level of somatic mutation. Besides, patients with high scores had unfavorable overall survival in the anti-PD-1 cohort, whereas the overall survival of high-score patients was better in the adoptive T cell therapy cohort. Our work highlights that m^6^A modification is closely related to immune infiltration in early-stage LUAD, which also contributes to the development of more effective immunotherapy strategies.

## Introduction

Lung adenocarcinoma (LUAD) is one of the most prevalent cancers around the world, accounting for approximately 40% of lung cancer patients (1). The study of LUAD has raised considerable concerns because it has a high rate of invasiveness and metastasis, which is the main cause of tumor-associated death (2, 3). Although with the rapid progress of various treatments, such as surgery, radiotherapy and chemotherapy, LUAD patients’ prognosis is still very poor (4–6). Some radiological approaches, such as low-dose computerized tomography (CT), are implemented to screen for LUAD and truly reduced the mortality of patients, but radiological approaches cannot benefit every patient and the diagnostic accuracy still has room for improvement (7, 8). Also, in the treatment of early-stage LUAD, chemotherapy could not reach the satisfactory efficacy among patients with negative driver gene mutation and the use of immunotherapy has remained largely unknown (9, 10). Thus, we need an in-depth investigation of detailed molecular mechanisms to identify patients with a high probability of death, which may contribute to the precise treatment of patients with early-stage LUAD.

N6-methyladenosine (m^6^A) has been regarded as the most common RNA modification, which primarily focuses on regulating splicing, translation and processing of the specific RNA. And it serves as a critically significant factor in diverse physiological and pathological processes (11–14). Generally speaking, m^6^A modification is regulated by three regulatory proteins: methyltransferases, binding proteins and demethylases (also known as writers, readers, and erasers) (15). Recent studies have indicated that m^6^A modification has a strong impact on the occurrence and metastasis of cancer, which suggests that a more comprehensive understanding of m^6^A modification’s detailed mechanism may benefit patients with cancer (14, 16). For example, Jin et al. have shown that m^6^A modification induced by METTL3 can increase YAP translation, thus promoting drug resistance and metastasis of non-small cell lung cancer (NSCLC) (17). However, in the early-stage LUAD, the exact roles of m^6^A modifications and their regulators remain largely unknown. Thus, the exploration of m^6^A modifications is urgently needed and may contribute to the development of m^6^A-based therapy.

Increasing evidence has revealed that multiple types of immune cells in the tumor microenvironment (TME) play a critically important role in tumorigenesis and metastasis (18). Furthermore, specific immune checkpoint inhibitors (ICIs), such as anti-PD-1/L1 and anti-CTLA-4, are widely applied in today’s immunotherapy and have been proven to be helpful in LUAD patients with specific immunophenotype (19–21). Thus, identifying particular characteristics of the TME may help to predict the immunotherapeutic responses, which could contribute to the development of more effective therapy in early-stage LUAD patients (22, 23). In addition, several studies have indicated a close relationship between m^6^A modification and immune infiltrating cells in the TME. Su et al. have revealed that deletion of FTO, an m^6^A regulatory protein, could inhibit the expression of LILRB4, thus dramatically attenuating the reprogram immune response of the leukemia stem cell. Due to the downregulation of FTO, leukemia cells were more sensitive to T cell cytotoxicity and immune evasion could be avoided (24). Also, Han et al. reported that the knockdown of YTHDF1 in dendritic cells could improve cross-priming of CD8+ T cells and cross-presentation of tumor neoantigens, suggesting YTHDF1 served as a critical biomarker in immunotherapy (25). However, we still lack a more comprehensive study focusing on all of the m^6^A regulatory proteins in early-stage LUAD. Therefore, exploring the relationship between m^6^A modification and immune infiltration may help us understand the regulation of the immune system and promote research in tumor immunotherapy.

In this study, we systematically analyzed the relationship between immune infiltrating levels and m^6^A modification clusters by using the genomic and transcriptomic data of 1230 early-stage LUAD patients. We have utilized nonnegative matrix factorization (NMF) clustering and identified three m^6^A clusters with different immune phenotypes, indicating m^6^A modification served as a non-negligible factor in affecting individual TME profiles. Furthermore, we also constructed the m^6^A-predictive score, which can be used to evaluate m^6^A modification, predict immune infiltrating levels and patients’ immunotherapeutic responses, suggesting its indispensable utility in clinical diagnosis and treatment.

## Materials and Methods

### The Collection of Available Datasets

We downloaded the genomic and clinical information of the early-stage LUAD from the GEO database (http://www.ncbi.nlm.nih.gov/geo/) and the TCGA database *via* UCSC Xena (https://xena.ucsc.edu/). According to the staging system of the American Joint Committee on Cancer (AJCC), LUAD of stage IA, IB, IIA and IIB could be defined as early-stage LUAD. 1230 patients were analyzed in this study, including patients from TCGA-LUAD (n = 374), GSE29013 (n = 22) (26), GSE30219 (n = 81) (27), GSE31210 (n = 226) (28), GSE37745 (n = 89) (29), GSE50081 (n = 127) (30) and GSE72094 (n = 311) (31). The baseline information of all of the early-stage LUAD patients was presented in **Table S2**. As for the TCGA-LUAD dataset, we obtained the copy number alteration data and the DNA methylation data (data of FMR1 was lack) from the cBioportal database (https://www.cbioportal.org/) and the somatic mutation data from the UCSC Xena. We analyzed the somatic mutation data using the “maftools” R package (version 2.6.05) (32). And we transformed the RNA-seq data of the TCGA-LUAD from the fragments per kilobase million (FPKM) format to transcripts per kilobase million (TPM) and log2(TPM + 1) format. Due to the same microarray platform (Affymetrix Human Genome U133 Plus 2.0 Array) used by GSE29013, GSE30219, GSE31210, GSE37745 and GSE50081, we obtained the raw CEL data and used the “gcrma” R package (version 2.62.0) to correct the background and normalize. Next, we used the ComBat function of the sva R package (version 3.38.0) to combine these five GSE datasets into a meta-GEO cohort (33). To increase the comparability among all the datasets, we utilized the scale transformation in the meta-GEO and GSE72094 cohorts before constructing the m6A-predictive score. Besides, we averaged expression values of genes that had multiple probe set signals.

### Consensus Clustering With NMF

We collected the 23 m^6^A regulators *via* retrieving previous publications correlated with m^6^A modification (**Table S1**) (13, 34–37). According to 23 m^6^A regulators’ expression levels, NMF clustering was performed to stratify distinct m^6^A clusters. The “NMF” R package (version 0.23.0) was utilized with the brunet algorithm and 50 nruns in this analysis (38). And we performed another NMF clustering according to the expression of overlapping differentially expressed genes (DEGs) with the lee method. The m^6^A related gene clusters (m^6^A cluster-R) were determined. According to the results of the NMF clustering (cophenetic, residuals, dispersion and rss coefficients), we chose the best number of clusters.

### GSVA, GSEA and GO/KEGG Enrichment Analysis

We used the “GSVA” R package (version 1.38.2) to perform Gene set variation analysis (GSVA), aiming to analyze different biological processes among all m^6^A related clusters (39). The Hallmarker gene set was used as the biological signatures and was obtained *via* the MSigDB database v7.2 (40). Gene Set Enrichment Analysis (GSEA) was applied *via* the “clusterProfiler” R package (version 3.18.1) and P.adjust < 0.05 was considered statistically significant (41). Gene Ontology (GO) and Kyoto Encyclopedia of Genes and Genomes (KEGG) analysis were also performed *via* the “clusterProfiler” R package (version 3.18.1). And the threshold of the GO analysis was P.adjust < 0.05 and the threshold of the KEGG analysis was P < 0.05 and P.adjust < 0.2.

### Estimation of Immune Cell Infiltration: ssGSEA, CIBERSORT and MCPcounter

We utilized different algorithms to evaluate the fraction of immune infiltrating cells, including single sample gene set enrichment analysis (ssGSEA), CIBERSORT and MCPcounter. The “GSVA” R package (version 1.38.2) was used to perform ssGSEA, which could calculate the fraction of twenty-eight immune cells of the TME. The estimated proportion of these immune infiltrating cells was characterized by a normalized score and shown in the heatmap. The CIBERSORT algorithm (http://cibersort.stanford.edu/) was designed to estimate the relative fraction of twenty-two immune cells (42). And we utilized the “MCPcounter” R package (version 1.2.0) to evaluate the abundances of two stromal cells and eight immune cells (43).

### Prediction of Immune Response: Immunophenoscore (IPS) and ESTIMATE

Immunophenoscore (IPS) serves as an essential factor in predicting response to anti-PD-1 and anti-CTLA-4 therapies. We calculated the IPS to investigate determinants of tumor immunogenicity, which also revealed cancer antigenomes and intratumoral immune features (44). ESTIMATE algorithm is generally utilized to infer the immune score and the stromal score, which is also useful to indicate the levels of immune infiltration (45). Based on the transcriptional profiles, we calculated the ESTIMATEScore, ImmuneScore and StromalScore to reveal different immune infiltrating levels of each cluster.

### DEGs Among Different m^6^A Clusters

Based on the results of the NMF clustering, three distinct m^6^A clusters were identified in the meta-GEO cohort. We then identified the DEGs between every two m^6^A clusters using the “limma” R package (version 3.46.0) (46). We calculated the differential expressed statistics *via* the lmFit and eBayes functions. We set |fold change| > 1 and P.adjust < 0.01 as the statistically significant threshold to identify DEGs. And we intersected three groups of DEGs to determine the overlapping DEGs among three different m6A clusters.

### Construction and Validation of the m^6^A-Predictive Score

Although the distinct m^6^A clusters were associated with prognosis and TME of patients with early-stage LUAD, it was not convenient to perform the NMF clustering in each independent cohort. Therefore, a more useful and reliable scoring system was required to analyze the prognosis and immune features of patients with early-stage LUAD. To begin with, we performed univariate Cox regression analysis using the “survival” R package (version: 3.2-10) to screen for the overlapping DEGs with prognostic value (overall survival) in the meta-GEO cohort. We defined P < 0.05 as the statistically significant threshold. The genes with an important prognostic impact were then analyzed with random forest (RF) algorithms using the “randomForestSRC” R package (version 2.10.1) and some genes were selected. Finally, we conducted the multivariate Cox regression analysis of these selected genes to screen for final genes and established the m^6^A-predictive score. The coefficients of the final genes were extracted from the multivariate Cox regression results. We used the following formula as the m^6^A-predictive score: score = ∑(Coefi * Expri), where n refers to the number of the key genes, Coef_i_ refers to the coefficient of gene_i_ and Expr_i_ refers to the expression level of gene_i_. We then calculated the m^6^A-predictive score of all samples and stratified patients into high- and low-score subgroups according to the median value of the m^6^A-predictive score. We also validated the efficacy of the m^6^A-predictive score in the TCGA and GSE72094 cohorts, respectively. We used the “survminer” R package (version 0.4.9) to get the Kaplan–Meier curves in these cohorts. And we evaluated the performance of this scoring system *via* the time-dependent receiver operating characteristic (ROC) curves using the “survivalROC” R package (version: 1.0.3).

### Establishment of the Nomogram

Using univariate and multivariate Cox regression analysis, we investigated whether our scoring system was an independent parameter among other clinical parameters, and the co-variates were composed of age and pathological stage. Then we utilized “rms” R package (version 6.1-1) to build a nomogram, which can predict the prognosis of patients with early-stage LUAD. Next, we used Calibration curves and the time-dependent ROC curves to evaluate this nomogram’ predictive accuracy. Also, we compared the concordance index among all the clinical parameters to analyze the discrimination of our nomogram.

### The Performance of m^6^A-Predictive Score in Immunotherapeutic Cohort

We further integrated two independent immunotherapy cohorts with genomic and clinical data to validate whether patients stratified by high and low m^6^A-predictive scores had significantly different clinical outcomes (overall survival). The X-tile software (version: 3.6.1) was utilized to identify the optimal cutoff value (47). A two-sided P value was used in the analysis. The data were downloaded from the TIDE database (http://tide.dfci.harvard.edu/) (48). The gene expression data were already normalized *via* the TIDE database. These cohorts mainly focused on the immunotherapy of patients with melanoma, which included anti-PD-1 antibody intervention in the study of Gide et al. (49) and the utilization of adoptive T cell therapy in the study of Lauss et al. (50).

### The Single-Cell Analysis for T Cells

We analyzed the T cells’ single-cell RNA-seq data of LUAD patients *via* http://lung.cancer-pku.cn/index.php, which contained 12 346 T cells from 14 treatment-naive NSCLC patients (51). The expression levels of selected m^6^A genes were normalized and sixteen clusters were identified, including two for regulatory T cells, seven for conventional CD4^+^ T cells and seven for CD8^+^ T cells. We used the boxplot and t-SNE plot to evaluate the associations between selected m^6^A genes and T cells populations.

### Statistical Analysis

We used R software (version 4.0.4) to conduct all of the statistical analyses. The analysis of the correlation was conducted *via* Spearman’s correlation method. We performed the Wilcoxon rank sum test (Mann-Whitney U-test) to compare the difference between the two groups. As for the difference among more than two groups, we used the Kruskal–Wallis H-test to evaluate the variance. Schoenfeld residuals was used to confirm the assumptions of the Cox proportional hazard models. *P* < 0.05 was considered as the statistically significant threshold. The tests used in each part of study were also presented in the figure.

## Results

### The Genetic Landscape of m^6^A Regulators in Early-Stage LUAD

**Figure 1:**
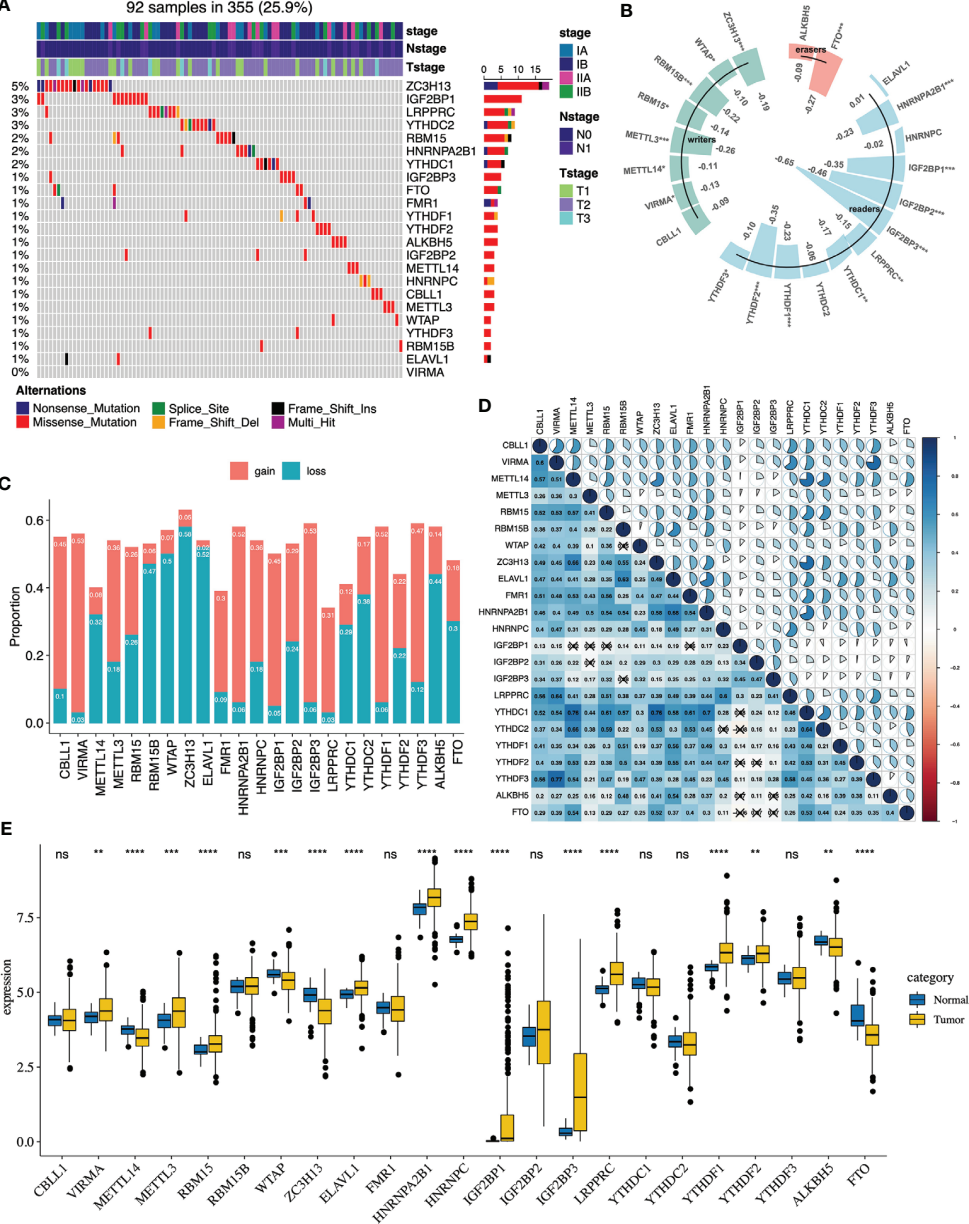
The genetic features of 23 m^6^A regulators in early-stage lung adenocarcinoma of the TCGA cohort. **(A)** The genetic mutations of 23 m^6^A regulators in 92 patients. **(B)** The correlation analysis between DNA methylation and 23 m^6^A regulators’ expression (*P < 0.05; **P < 0.01; ***P < 0.001). **(C)** The CNA landscape of 23 m^6^A regulators. Gain represents gain and high level amplification, loss represents homozygous deletion and hemizygous deletion. **(D)** The correlation analysis between the 23 m^6^A regulators *via* the Spearman correlation method. **(E)** The m^6^A regulators expression between normal and tumor groups (*P < 0.05; **P < 0.01; ***P < 0.001; ****P < 0.0001). ns, not significant.

The flowchart of our study is presented in **Figure S1**. The somatic mutations of 23 m^6^A regulators were infrequent in early-stage LUAD. 92 of the 355 (25.9%) samples had the somatic mutations of m^6^A regulators, which primarily included missense mutations and nonsense mutations. The results revealed that the top three genes with the highest mutation were ZC3H13 (5%), IGF2BP1 (3%) and LRPPRC (3%) (**Figure 1A**). Then, we explored the relationship between m^6^A regulators’ expression and DNA methylation (**Table S3**). All of the significant correlations between m^6^A regulators’ expression and DNA methylation were negative. IGF2BP1, IGF2BP2, and IGF2BP3 were the top three genes whose expression was closely related to the DNA methylation level (**Figure 1B**). The copy number alterations of the m^6^A regulators were prevalent, which included copy number gains and losses. VIRMA, IGF2BP3, YTHDF1 displayed prevalent copy number gains, while ZC3H13, ELAVL1 and WTAP showed widespread copy number losses (**Figure 1C**). Compared with normal samples, we found that VIRMA, METTL3, RBM15, ELAVL1, HNRNPC, HNRNPA2B1, IGF2BP1, IGF2BP3, YTHDF1, YTHDF2, LRPPRC were highly expressed in tumors, while METTL14, ZC3H13 and FTO were mainly downregulated (**Figure 1E**). We then evaluated the correlation of the expression levels among the m^6^A regulators. According to the Spearman correlation analysis, we found that most of m^6^A regulators revealed a significant inverse correlation with the other, such as HNRNPA2B1, YTHDF1 and HNRNPC (**Figure 1D** and **Table S4**). Additionally, we utilized univariate and multivariate Cox regression to evaluate m^6^A regulators’ prognostic value (overall survival). The forest plot suggested that RBM15 was highly associated with patients’ overall survival and could be recognized as a protective factor, while ALKBH5 was negatively correlated with the patient’s prognosis (**Figures S2A, B**).

### Identification of Specific Phenotypes Based on m^6^A Regulators

To stratify patients into different m^6^A phenotypes, we performed the NMF algorithm according to 23 m^6^A regulators’ expression levels (**Figure S3A, B**). Three m^6^A phenotypes were identified in the meta-GEO cohort, including 120 patients in m^6^A-C1, 230 patients in m^6^A-C2 and 195 patients in m^6^A-C3. Then, we conducted a log-rank test and the Kaplan-Meier curves of the meta-GEO cohort revealed that m^6^A-C2 had the best prognosis, whereas m6A-C1 was related to unfavorable prognosis (**Figure S4C**). And similar results were shown in the Kaplan-Meier curves of the TCGA cohort (**Figure S4D**). The expression patterns of these three clusters were aberrantly different. The expression level of YTHDF3 was increased in patients of m^6^A-C3; The expression levels of IGF2BP1, IGF2BP3 were predominantly elevated in patients of m^6^A-C1; The expression level of IGF2BP2, LRPPRC, WATP, YTHDF3 were relatively increased in patients of m^6^A-C2 (**Figures S3C, D** and **Table S6**). We further conducted multivariate Cox regression analysis to determine the correlation of the m^6^A clusters and patients’ clinical outcomes. The results indicated that m^6^A clusters were correlated with patients’ OS especially in the TCGA cohort (m^6^A-C2 *vs*. m^6^A-C1, HR = 0.529 [95%CI = 0.308 - 0.908], P = 0.0209; **Figure S4**).

### The Expression Levels of m^6^A Genes in Exhausted T Cells in LUAD

We have analyzed the expression levels of 23 m^6^A genes in different T cells of LUAD *via* a single-cell database. The results indicated that CBLL1 and WTAP served as two significant factors in T cells infiltration. The expression levels of WTAP were relatively higher in CD4-C2-ANXA1, CD4-C7-CXCL13, CD4-C9-CTLA4 and CD8-C6-LAYN populations than others, indicating CD8^+^ T cells and conventional CD4^+^ T cells (**Figure S9A**). The expression levels of CBLL1 were relatively higher in CD4-C2-ANXA1 and CD4-C8-FOXP3 populations than others, indicating conventional CD4^+^ T cells and regulatory T cells (**Figure S9C**). According to the t-SNE plot, the T cells enrichment regions with high CBLL1 and WTAP expression were also highly overlapped with the above clusters (**Figures S9B, D, E**).

### The Correlation of m^6^A Phenotypes With Immune Infiltration

**Figure 2:**
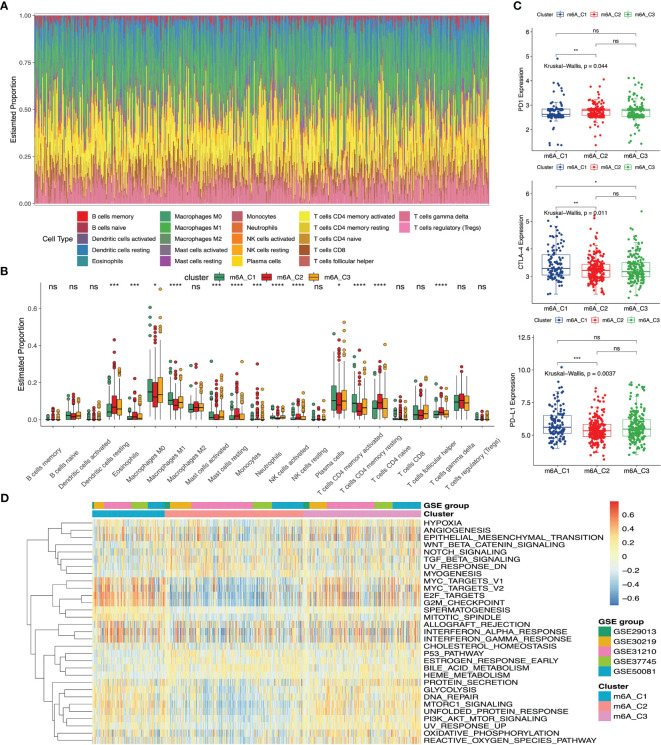
The immune and biological landscape of three different clusters in meta-GEO cohort. **(A)** The results of the CIBERSORT analysis reveals the estimated proportion of different immune infiltrating cells. **(B)** The results of the CIBERSORT analysis reveals the estimated proportion of multiple immune infiltrating cells in three m^6^A clusters. Kruskal-Wallis H test was applied to compare the difference among three clusters (*P < 0.05; **P < 0.01; ***P < 0.001, ****P < 0.0001; ns, not significant). The estimated proportion of each immune cell was symbolized by the scattered dots. The median proportion was symbolized by the thick line. The interquartile range was represented by the range between the top and bottom of each box. **(C)** The difference of PD1, CTLA-4 and PD-L1 expression levels among three m^6^A clusters. **(D)** GSVA results were presented in the meta-GEO cohort among three clusters. The sample annotations of the heatmap were the composition of different GEO cohorts.

We utilized the Hallmarker gene set to perform the GSVA enrichment analysis, which revealed different biological processes. Some tumor-related targets (MYC, E2F and G2M) and tumor-related biological processes (angiogenesis, epithelial-mesenchymal transition (EMT) and hypoxia) were enriched in m^6^A-C1. On the contrary, m^6^A-C2 were relatively enriched pathways of cancer development and progression, including Wnt-β-catenin, Notch and TGF-β signaling pathways. And m^6^A-C3 exhibited the GSVA scores between m^6^A-C1 and m^6^A-C2 in the above gene sets (**Figure 2D**). To determine the relationship between different m^6^A phenotypes and immune infiltrating cells, we conducted the CIBERSORT algorithm among three m^6^A clusters. The fraction of immune infiltrating cells was presented in **Figure 2A**. We also compared the fraction of immune cells among these three m^6^A clusters. The results indicated that the proportion of macrophages M0, macrophages M1, plasma cells and activated memory CD4 T cells were the highest in m^6^A-C1, the proportion of dendritic cells resting, eosinophils, monocytes, mast cells resting, T cells CD4 memory resting, T cells follicular helper and NK cells activated were the highest in m^6^A-C2 and the proportion of mast cells activated were the highest in m^6^A-C3 (**Figure 2B**). To further evaluate the clinical significance of these three m^6^A phenotypes, we also analyzed the expression of different biomarkers (PD-1, PD-L1 and CTLA-4) in the immunotherapy (52, 53). The results revealed that PD-L1 and CTLA-4 expression were markedly elevated in m^6^A-C1, while PD-1 expression was relatively increased in m^6^A-C2 (**Figure 2C**).

We then utilized Spearman’s correlation analyses to investigate the relationship between different immune infiltrating cells and m^6^A regulators. Our results revealed that the expression of LRPPRC, VIRMA, YTHDF2, YTHDC1, HNRNPC, METTL14 and METTL3 were negatively correlated with immune infiltration, whereas WTAP expression was positively related to immune infiltration (**Figure S5A**). LRPPRC was negatively correlated with infiltrating levels of immune cells, including type 1 T helper cell, T follicular helper cell, macrophage, effector memory CD8 T cell and activated dendritic cell. Thus, we divided the patients into two subgroups according to the high- and low-expression level of LRPPRC to analyze its role in early-stage LUAD. We used the CIBERSORT algorithm to explore the difference of immune infiltration between low- and high-LRPPRC. Our results implied that infiltrating levels of mast cells resting, dendritic cells activated, T cells follicular helper and T cells CD4 memory activated were relatively elevated in the high-LRPPRC group, whereas T cells CD8, mast cells activated, and plasma cells were the opposite (**Figure S5B**). Furthermore, we analyzed the immune-related scores between the two groups, which indicated that ImmuneScore, ESTIMATEScore and StromalScore were higher in the high-LRPPRC group (**Figure S5C**). And we evaluated biomarkers’ expression in immunotherapy. We found LRPPRC expression was negatively associated with the expression of PD-1, PD-L1 and CTLA-4 (**Figure S5D**). Besides, we performed the GSEA analysis to evaluate related gene sets of the low- and high-LRPPRC subgroups. Our results implied that patients in the low-LRPPRC enriched genes of mRNA processing, RNA splicing and mRNA processing, and patients in the high-LRPPRC enriched genes of *Staphylococcus aureus* infection, hematopoietic cell lineage and complement and coagulation cascades (**Figure S5E**). In a word, we hypothesized that LRPPRC could impede the activation of immune cells (like CD8 T cells) or mRNA processing to regulate cancer development and progression.

### The DEGs Among Three m^6^A Phenotypes

**Figure 3:**
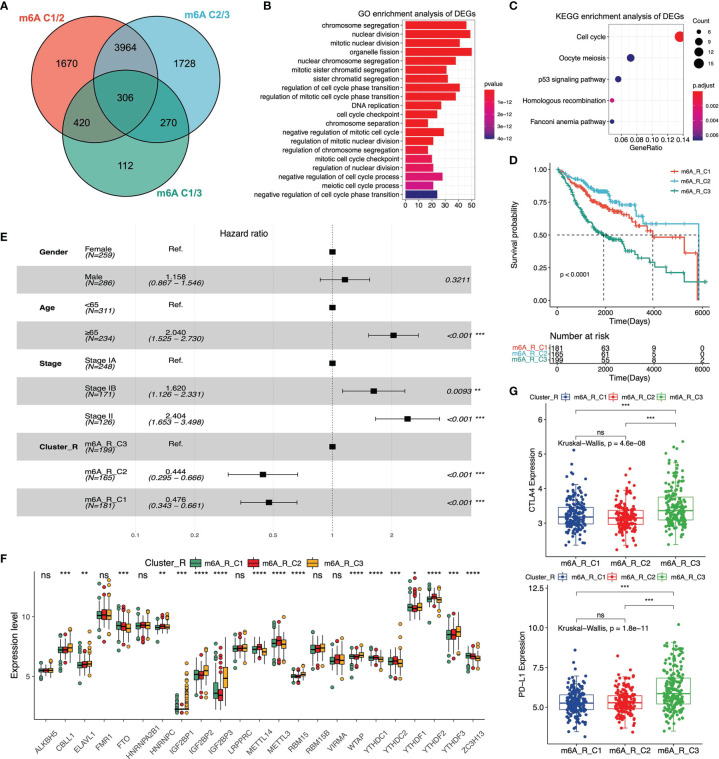
Analysis of differentially expressed genes (DEGs) among three m^6^A clusters and enrichment analysis in meta-GEO cohort. **(A)** The Venn diagram reveals the m^6^A correlated differentially expressed genes. **(B)** GO enrichment analysis of the DEGs among three clusters. **(C)** KEGG enrichment analysis of the DEGs among three clusters. **(D)** The Kaplan-Meier curves of three m^6^A gene-related clusters *via* Log-rank test. **(E)** Multivariate Cox regression analysis of clinical parameters among three m^6^A gene-related clusters. 95% confidence interval was symbolized by the horizontal line. Hazard ratio (HR) was symbolized by the vertical dotted line. **(F)** The 23 m^6^A regulators’ expression among three m^6^A gene-related clusters. **(G)** The expression levels of CTLA-4 and PD-L1 among three m^6^A gene-related clusters. *P < 0.05; **P < 0.01; ***P < 0.001; ****P < 0.0001; ns, not significant.

To determine the extensive role of these three m^6^A clusters stratified by 23 m^6^A regulators’ expression, we further analyzed the DEGs across m^6^A-C1, m^6^A-C2 and m^6^A-C3 in the meta-GEO cohort. 306 DEGs were regarded as m^6^A-correlated signatures and utilized for later analysis. The Venn diagram was used to reveal the overlapping DEGs among the three m^6^A clusters (**Figure 3A**). Next, we carried out the GO and KEGG enrichment analysis of these overlapping DEGs to evaluate relevant biological processes and pathways. The results indicated that this m^6^A-correlated signature was closely related to some biological processes, including chromosome segregation, nuclear division and regulation of cell cycle phase transition (**Figure 3B**). And m^6^A-correlated signature was closely related to the pathways, including p53 signaling, oocyte meiosis and cell cycle pathway (**Figure 3C**). According to the overlapping DEGs, we utilized the NMF clustering analysis and stratified patients in the meta-GEO cohort into three distinct clusters (**Figures S6A, B**). These three clusters were defined as m^6^A-R-C1, m^6^A-R-C2 and m^6^A-R-C3, which showed different clinical parameters. The heatmap revealed that patients with pathological stage IA were mainly classified by m^6^A-R-C2, whereas patients with pathological stage II were mostly represented by m^6^A-R-C3 (**Figure S6C** and **Table S5**). We then evaluated the overall survival of patients in the three cluster-Rs *via* multivariate Cox regression analysis and the log-rank test. Kaplan-Meier curves showed that patients in m^6^A-R-C2 had the best prognosis, whereas patients in m^6^A-R-C3 had the worst prognosis (**Figure 3D**). The results revealed that age, pathological stage and cluster-R were associated with patients’ OS (m^6^A-R-C2 *vs*. m^6^A-R-C3, HR = 0.444 [95%CI = 0.295 - 0.666], P < 0.001; m^6^A-R-C1 *vs*. m^6^A-R-C3, HR = 0.476 [95%CI = 0.343 - 0.661], P < 0.001, **Figure 3E**). We further evaluated m^6^A regulators’ expression among three cluster-Rs and determined that most of the m^6^A regulators’ expression varied in different cluster-Rs (**Figure 3F**). Moreover, we analyzed the immune landscape of the three cluster-Rs. We performed the ssGSEA analysis to evaluate the proportion of twenty-eight immune cells in these three m^6^A gene-related phenotypes. We found that activated CD4 T cells, memory B cells were relatively enriched in m^6^A-R-C3 (**Figure S4E**). We then utilized the ESTIMATE algorithm to figure out the differences among the three cluster-Rs. The results indicated that m^6^A-R-C2 exhibited the lowest ImmuneScore, ESTIMATEScore and StromalScore, suggesting that m^6^A-R-C2 was rarely related to immunity (**Figure S6D**). Also, we found that CTLA-4 and PD-L1 expression were lowest in m^6^A-R-C2 and highest in m^6^A-R-C3, which also suggested different immune features across three cluster-Rs (**Figure 3G**).

### Establishment of the m^6^A-Predictive Score and Nomogram

**Figure 4:**
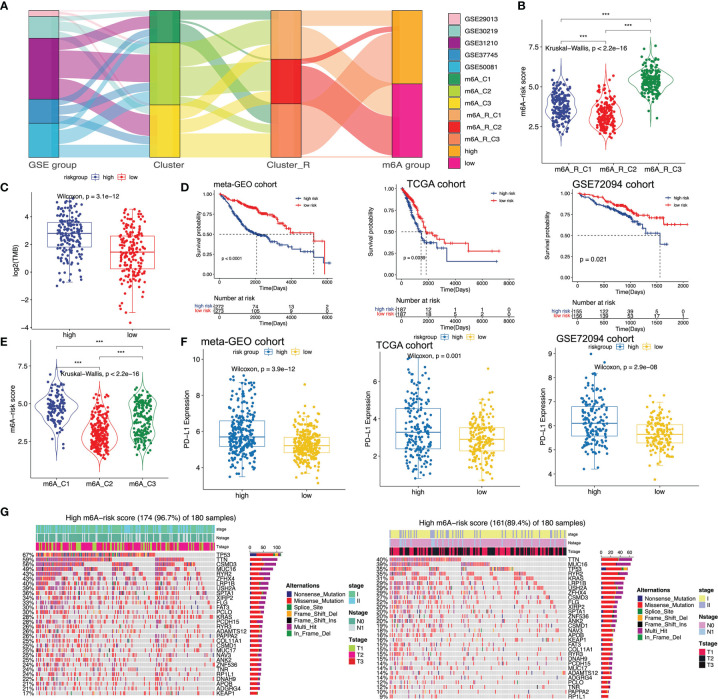
Establishment of m^6^A-predictive score and evaluation its correlated clinical landscapes. **(A)** Sankey diagram of different GSE groups with various m^6^A clusters, m^6^A cluster-Rs and m^6^A-predictive score. **(B)** Comparison of m^6^A-predictive score among three clusters in the meta-GEO cohort (*P < 0.05; **P < 0.01; ***P < 0.001). **(C)** Comparison of tumor mutation burden (TMB) between low and high m^6^A-score groups in TCGA cohort. **(D)** Kaplan-Meier curves of low and high m^6^A-score groups in meta-GEO, TCGA and GSE72094 cohorts *via* Log-rank test. **(E)** Comparison of m^6^A-predictive score among three clusters in the meta-GEO cohort (*P < 0.05; ** P< 0.01; ***P < 0.001). **(F)** Distribution of PD-L1 expression levels in different m^6^A-score groups of meta-GEO, TCGA and GSE72094 cohorts. **(G)** Mutational features of the top mutated genes in low and high m^6^A-score groups in the TCGA cohort. The sample annotations were shown in the upper part of the diagram, including stage, Tstage and Nstage.

Although m^6^A-related clusters and cluster-Rs can stratify early-stage LUAD patients into distinct groups correlated with different prognoses, this predictive method was not efficient to predict patients in the individual cohort. Thus, a more accurate and consistent predictive model based on m^6^A-related clusters and cluster-Rs was needed. And we established the m^6^A-predictive score to classify early-stage LUAD patients, which was of great importance. The m^6^A-predictive score was established based on five key genes selected *via* univariate Cox regression analysis (**Table S7**), random forest analysis and multivariate Cox regression analysis (**Table S8**) (m^6^A-predictive score = -3.2370611 * Expr_LRIG1_ + 0.3936202 * Expr_CTSV_ + 0.5548459 * Expr_KIF20A_ + 0.7905488 * Expr_ATP13A3_ - 0.4148747 * Expr_TMPRSS2_). We used the alluvial diagram to illustrate the connection among GSE groups, m^6^A-related clusters, m^6^A-related cluster-Rs and m^6^A-predictive score (**Figure 4A**). We identified patients in m^6^A-C1 were more likely to have a higher m^6^A-predictive score, while patients in m^6^A-C2 were more likely to have a lower m^6^A-predictive score. The above findings were also illustrated by a violin diagram (**Figure 4E**). Also, we found that patients in the m^6^A-R-C3 had the highest m^6^A-predictive score among these three cluster-Rs (**Figure 4B**). We then divided the patients into low- and high-score subgroups and determined the efficacy of the m^6^A-predictive score (**Figure S7B**). In the meta-GEO cohort, Kaplan-Meier curves implied that a higher m^6^A-predictive score was related to a worse prognosis of early-stage LUAD patients (**Figure 4D**, left panel). And we utilized the time-dependent ROC curves to evaluate the predictive efficacy of the m^6^A-predictive score in the meta-GEO cohort, which revealed that the 1-, 3- and 5-year AUCs were 0.67, 0.71 and 0.75 (**Figure S7A**). Besides, we also analyzed the relationship between PD-L1 expression and m^6^A-predictive score in the meta-GEO cohort. We observed that PD-L1 expression was remarkedly elevated in the high m^6^A-score group (**Figure 4F**, left panel).

Next, we validated the m^6^A-predictive score with two independent cohorts, the TCGA cohort and the GSE72094 cohort. We first calculated the m^6^A-predictive score and divided each cohort into low- and high-score subgroups in TCGA (**Figure S7D**) and GSE72094 cohorts (**Figure S7F**). Log-rank test was then utilized to determine the correlation of m^6^A-predictive score and the clinical outcomes in both cohorts. We found that in the TCGA and GSE72094 cohorts, the increase of predictive score was related to poor clinical outcomes (**Figure 4D**, middle and right panel). Also, we evaluated the efficacy of the m^6^A-predictive score and the results revealed that 1-, 3- and 5-year AUCs were 0.6, 0.61 and 0.63 in the TCGA cohort (**Figure S7C**), 0.69, 0.63 and 0.84 in the GSE72094 cohort, indicating this scoring system’s 5-year predictive efficacy was the highest (**Figure S7E**). We also found that PD-L1 expression was mainly increased in the high-score group (**Figure 4F**, middle and right panel). Recent studies have found that TMB can play an essential role in predicting the response of immunotherapy (54). Thus, we further explored the correlation of m^6^A-predictive score and TMB in the TCGA cohort. Our results revealed that patients of the high-score group had higher TMB (**Figure 4C**). Besides, we analyzed the top mutational genes in these two subgroups respectively. The mutational feature indicated that genes in the high-score group had higher mutational rates than the other (**Figure 4G**). The top three genes in the high-score group were TP53 (67%), TTN (59%) and CSMD3 (56%), while those in the other group were TTN (40%), MUC16 (39%) and TP53 (35%). In a word, we comprehensively evaluated the interaction between m^6^A-predictive score and somatic mutations.

We then integrated clinical parameters and m^6^A-predictive score to build a nomogram, which aimed to elevate the accuracy and reliability of the predictive model in early-stage LUAD. Univariate and multivariate Cox regression models were utilized in the meta-GEO cohort, and the results revealed that age, stage and m^6^A-predictive score were remarkedly related to patients’ OS (**Figure S8A**). Then we built the nomogram according to the above three clinical characteristics (**Figure S8C**). And we compared the C-index among all of the selected clinical parameters, which indicated that the nomogram had the highest C-index (**Figure S8B**). Additionally, the calibration plots revealed the great concordance between the prognosis and our nomogram, indicating our nomogram served as an essential factor to predict clinical outcomes of early-stage LUAD patients (**Figure S8E**). Furthermore, the time-dependent ROC curves were utilized to evaluate the accuracy of our nomogram, which showed that 1-, 3- and 5-year AUCs were 0.68, 0.74 and 0.75, respectively (**Figure S8D**).

### Application of m^6^A-Predictive Score in Predicting the Immunotherapeutic Effect

**Figure 5:**
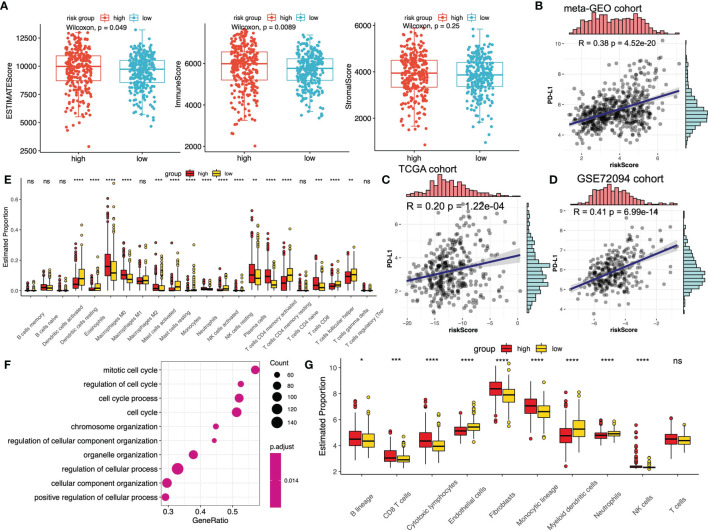
The relationship between m^6^A-predictive score and immune infiltration. **(A)** Distribution of different immune scores (ESTIMATEScore, ImmuneScore and StromalScore) in low and high m^6^A-score groups. **(B–D)** The correlation analysis of PD-L1 expression levels and m^6^A-predictive score *via* Spearman correlation method in meta-GEO cohort, TCGA cohort and GSE72094 cohort. **(E)** The estimated fraction of different immune infiltrating cells calculated by CIBERSORT algorithm in different m^6^A-score groups (*P < 0.05; **P < 0.01; ***P < 0.001; ****P < 0.0001). **(F)** GSEA enrichment analysis (GO) of the top differentially expressed genes in the group with a high m^6^A-predictive score. **(G)** The estimated fraction of different immune infiltrating cells calculated by MCPcounter algorithm in different m^6^A-score groups. ns, not significant.

To explore the detailed relationship between m^6^A-predictive score and tumor immunity, we compared the immune scores between low- and high-score subgroups in the meta-GEO cohort. The results indicated that ESTIMATEScore and ImmuneScore of the high-score group were predominantly higher, while there was no difference of StromalScore between the two groups (**Figure 5A**). We then calculated the fraction of immune infiltrating cells between these two subgroups in the meta-GEO cohort *via* the different algorithms. Using the CIBERSORT algorithms, the results implied that the fraction of macrophages M0, macrophages M1, mast cells activated, neutrophils, plasma cells, T cells CD8 and T cells CD4 memory activated were relatively higher in the high-score group, whereas dendritic cells resting, eosinophils, monocytes, mast cells resting, T cells gamma delta, T cells follicular helper, T cells CD4 memory resting, NK cells resting, and NK cells activated were lower (**Figure 5E**). And the results of the MCPcounter algorithms implied that the fraction of B lineage, CD8 T cells, cytotoxic lymphocytes, fibroblasts, monocytic lineage and NK cells were higher in the high-score group, whereas the fraction of endothelial cells, neutrophils, myeloid dendritic cells were lower (**Figure 5G**). Besides, we performed the GSEA enrichment analysis of the high-score subgroup, which revealed that mitotic cell cycle, regulation of cell cycle and cell cycle process were the top three biological processes enriched (**Figure 5F**).

**Figure 6:**
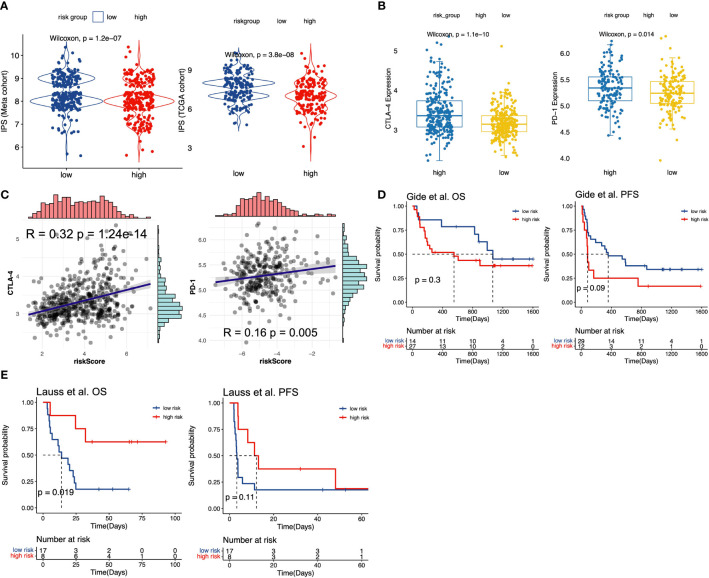
The value of m^6^A-predictive score to predict patients’ clinical outcomes with immunotherapy. **(A)** Comparison of IPS scores between low and high m^6^A-score groups in meta-GEO and TCGA cohorts. **(B)** The distribution of CTLA-4 expression levels between low and high m^6^A-score groups in meta-GEO cohort. The distribution of PD-1 expression levels between low and high m6A-score groups in GSE72094 cohort. **(C)** The Spearman correlation analysis of CTLA-4 and predictive score in meta-GEO cohort. The Spearman correlation analysis of PD-1 and predictive score in GSE72094 cohort. **(D)** Kaplan-Meier curves for low and high m^6^A-score groups in Gide et al. (49) cohort. **(E)** Kaplan-Meier curves for low and high m^6^A-score groups in Lauss et al. (50) cohort.

Immune checkpoint inhibitors (ICIs) treatment was one of the emerging immunotherapies, which was widely used in clinical practice (55). Some prevalent ICIs have been already utilized to treat cancer patients, including PD-1/L1 and CTLA-4 (21, 56). In addition, some recent studies have indicated that IPS could be regarded as a novel predictor to predict immunotherapeutic responses. Thus, we first performed the correlation analysis to evaluate the correlation of m^6^A-predictive score and PD-L1 expression. Our study found that PD-L1 expression was strongly related to m^6^A-predictive score in meta-GEO cohort (**Figure 5B**, R = 0.38, *P* = 4.52e-20), TCGA cohort (**Figure 5C**, R = 0.20, *P* =1.22e-04) and GSE72094 cohort (**Figure 5D**, R = 0.41, *P* = 6.99e-14). Also, we analyzed the correlation of other two ICIs (PD-1 and CTLA-4) and the m^6^A-predictive score. Our results implied that CTLA-4 expression was increased in the high-score group of the meta-GEO cohort and PD-1 expression was relatively higher in the GSE72094 cohort (**Figure 6B**). And m^6^A-predictive score was correlated with CTLA-4 (R = 0.32, *P* = 1.24e-14) and PD-1 expression (R = 0.16, *P* = 0.005) in meta-GEO and GSE72094 respectively (**Figure 6C**). We then evaluated the distribution of the IPS score between these two subgroups, which revealed that the IPS score was predominantly higher in the low-score group in the meta-GEO and TCGA cohorts (**Figure 6A**). Furthermore, due to the correlation of immune response and m^6^A-predictive score, we applied our m^6^A-predictive score to evaluate its predictive value in an anti-PD-1 cohort (49) (study of Gide et al.) and an adoptive T cell therapy cohort (50) (study of Lauss et al.). Based on the log-rank test, our results revealed that patients with low m^6^A-predictive scores exhibited prolonged OS and PFS in the anti-PD-1 cohort (**Figure 6D**). On the contrary, the prognosis of patients with the low m^6^A-predictive score was poor in the adoptive T cell therapy cohort (**Figure 6E**). In conclusion, the above findings indicated that our m^6^A-predictive score was significantly related to immunotherapies and could serve as a crucial factor in predicting patients’ prognoses.

## Discussion

Abundant evidence revealed that m^6^A modification served as a critically essential factor in tumor immunity and could regulate malignant behaviors *via* the complex interaction among different m^6^A regulatory proteins (57, 58). Previous publications have investigated the role of several m^6^A regulators in the TME, but no study has systematically explored the whole TME characteristics recognized by diverse m^6^A regulators in early-stage LUAD. Thus, classifying different m^6^A modification clusters in the TME could expand our knowledge of the more comprehensive association between anti-tumor immunotherapy and m^6^A modification, which may promote the development of more efficient and reliable immunotherapy strategies.

In the present study, we have identified three distinct m^6^A clusters according to 23 m^6^A regulators’ expression levels, which were classified by different biological processes. Our results implied that the m^6^A-C1 was classified through multiple tumor-related biological processes (angiogenesis, EMT and hypoxia) and biomarkers (MYC and E2F); m^6^A-C2 was classified through several pathways correlated with tumorigenesis and progression, such as Wnt, TGF-beta and Notch pathways. Emerging evidence indicated that TME could play an essential role in the occurrence and development of tumors, which can also affect the immunotherapeutic responses (18, 59). Moreover, studies have shown that patients with more abundance of dendritic cells (60), NK cells (61) and T follicular helper cells (62) were more likely to respond to immunotherapy with ICIs. The m^6^A-C2 was mostly identified with its high infiltrating levels of the above immune cells, suggesting its potential strengths in predicting immunotherapeutic responses. And the m^6^A-C2 was also proven to have the best prognosis among three m^6^A modification clusters. Previous publications have demonstrated that the activation of some targets (MYC and E2F) was closely related to tumorigenesis and metastasis (63, 64). In addition, studies have revealed that EMT and hypoxia were related to poor prognosis and may be highly correlated with TME (65, 66). These biological processes and some specific immune infiltrating cells were enriched in m^6^A-C1, and the survival analysis showed that patients of m^6^A-C1 had unfavorable prognoses. Integrating the biological processes and TME characteristics of different m^6^A clusters, our stratification was proven to be reliable and may foster the research of immunotherapy in early-stage LUAD.

Next, we analyzed the biological processes of the overlapping DEGs among the three m^6^A modification clusters, and the results revealed that RNA segregation and nuclear division were enriched, indicating the overlapping DEGs could be considered as an m^6^A-correlated signature. Three m^6^A cluster-Rs were then identified based on the overlapping DEGs, which also exhibited a strong correlation with prognosis and TME characteristics. The results revealed that m^6^A modification could serve as a significant factor in classifying patients with different TME features. The m^6^A-R-C2 was rarely related to immunity and had the best clinical outcomes, whereas m^6^A-R-C3 was the opposite. However, due to the heterogeneity of each patient, using m^6^A clusters or cluster-Rs to stratify patients into independent groups was not convenient and effective. Therefore, we constructed a scoring system (m^6^A-predictive score) to accurately quantify m^6^A modified subgroups. The m^6^A-C1 with relatively low immune infiltrating levels had a higher m^6^A-predictive score, while the m^6^A-C1 with abundant immune infiltrating cells had a lower m^6^A-predictive score. We further evaluated the exact role of the m^6^A-predictive score, which showed that this score could be an effective prognostic predictor in the training and validation cohorts of early-stage LUAD. In addition, our results implied a strong relationship between this score and TMB, somatic mutational rate, immune-related score and immune response predictors (IPS, PD-1, PD-L1 and CTLA-4). The findings suggested m^6^A-predictive score was a robust and reliable scoring system, which can be used to define the m^6^A modification subgroups in independent cohorts, and the scoring system was also associated with specific immune features. Moreover, we used this scoring system to evaluate its predictive efficacy in two immunotherapy cohorts. Interestingly, our study found that patients’ clinical outcomes with low m^6^A-predictive scores were favorable in the anti-PD-1 cohort, while the prognosis was poor in the adoptive T cell therapy cohort. The results implied the different performance of our m^6^A-predictive score in different kinds of immunotherapy cohorts, suggesting its precision of predicting immunotherapeutic responses.

Due to LRPPRC being negatively correlated with most of the immune infiltrating cells, we then analyzed its role in modulating the TME. Previous publications have revealed that LRPPRC was involved in multiple physiological and pathological processes, such as energy metabolism (67). As one of the m^6^A regulatory proteins, LRPPRC expression increased in diverse cancer tissues but decrease in normal tissues (67). To be specific, Tian et al. revealed that upregulation of LRPPRC was associated with growth and invasion and was related to poor prognosis in LUAD (68). In our study, we divided patients into two subgroups according to LRPPRC expression and compared the immune landscapes between the low- and high-expression subgroups. We found that immune-related scores and immune checkpoints expression were relatively lower in the high-expression group, which could offer strong evidence that LRPPRC expression was negatively associated with immune infiltration in the TME. Due to the lack of studies of the relationship between LRPPRC and the TME, our work may shed new light on the research of this LRPPRC and provide a new understanding of m^6^A regulators’ role in the TME.

Somatic mutational genes play a crucial role in tumorigenesis and progression, which are highly related to the diagnosis, treatment and prognosis in various cancers (69, 70). Here, we evaluated the difference of somatic mutation genes between the low- and high-score subgroups. Our results implied that the mutational rates of TP53, TNN and CSMD3 were the top three in the high-score group, whereas TNN, MUC16 and TP53 were the top three mutational genes in the low-score group. Li et al. have reported that MUC16 mutations were strongly correlated with immune-related pathways in gastric cancer (71). Specifically, MUC16 mutations could increase the infiltrating levels of cytotoxic T lymphocytes in the TME (72). And TTN was also proven to be an essential factor in predicting responses of immunotherapy with ICIs (73). TP53 mutations were ubiquitous in various cancers, which could predict responses of anti-PD-1 immunotherapy in LUAD (74, 75). The above gene mutations related to the m^6^A-predictive score were significantly correlated with tumor immunity, indicating the complex relationship between TME characteristics and m^6^A modification.

This research provided a novel insight into the interaction of m^6^A modification and TME characteristics in the early-stage LUAD. However, some limitations still existed in the work. First of all, the results of our bioinformatical research needed to be verified by using clinical trials with comprehensive clinical information. Secondly, to maintain the efficacy and reliability of our scoring system, newly identified m^6^A regulatory proteins were supposed to be integrated into our model in the future. Moreover, different kinds of appropriate immunotherapy cohorts of early-stage LUAD were needed to further validate the accuracy of the m^6^A-predictive score. In addition, our study was a retrospective study, and we need a prospective clinical trial of early-stage LUAD with immunotherapy to further strengthen our results.

In the present study, we systematically revealed the extensive role of 23 m^6^A regulators in TME by integrating 1230 early-stage LUAD patients. We found that different phenotypes classified by the m^6^A modification have distinct immune characteristics, indicating the strong interaction between tumor immunity and m^6^A modification in early-stage LUAD. Furthermore, a scoring system was established to evaluate the m^6^A modification phenotypes and the immune infiltration in individual cohorts. The investigation of m^6^A modification in early-stage LUAD could contribute to a better understanding of TME features and facilitate the development of more promising and potent immunotherapy in the future.

## Data Availability Statement

All datasets presented in this study are included in the article/**Supplementary Material**. GEO data was downloaded from the GEO database (https://www.ncbi.nlm.nih.gov/gds/) under the accession number(s) GSE29013, GSE30219, GSE31210, GSE37745, GSE50081 and GSE72094. TCGA RNA-seq data were downloaded from the TCGA database via the UCSC Xena (https://xena.ucsc.edu/) under the accession number(s) LUAD-FPKM. The copy number alteration data and the DNA methylation data of the TCGA cohort were downloaded from the cBioportal database (https://www.cbioportal.org/). The immunotherapy data were downloaded from the TIDE database (http://tide.dfci.harvard.edu/) under the accession name “Gide et al., Cancer Cell 2019” and “Lauss et al., Nat Commun 2017”. All of the data can also be accessed by contacting with the corresponding authors.

## Author Contributions

BZ and SG designed and supervised the study. BZ analyzed the data and wrote the original draft. SG edited the draft. All authors contributed to the article and approved the submitted version.

## Funding

The study was funded by Institutional Fundamental Research Funds (2018PT32033), the Ministry of Education Innovation Team Development Project (IRT-17R10) and ETHICON·Excellent in surgery grant (2018-011-ZZ).

## Conflict of Interest

The authors declare that the research was conducted in the absence of any commercial or financial relationships that could be construed as a potential conflict of interest.

## Publisher’s Note

All claims expressed in this article are solely those of the authors and do not necessarily represent those of their affiliated organizations, or those of the publisher, the editors and the reviewers. Any product that may be evaluated in this article, or claim that may be made by its manufacturer, is not guaranteed or endorsed by the publisher.

## Abbreviations

m^6^A, N6-methyladenosine; LUAD, lung adenocarcinoma; NMF, non-negative matrix factorization; NSCLC, non-small cell lung cancer; TME, tumor microenvironment; ICIs, immune checkpoint inhibitors; IFN-γ, interferon-gamma; FPKM, fragments per kilobase million; TPM, transcripts per kilobase million; DEGs, differentially expressed genes; GSVA, Gene set variation analysis; GSEA, Gene Set Enrichment Analysis; GO, Gene Ontology; KEGG, Kyoto Encyclopedia of Genes and Genomes; ssGSEA, Single sample gene set enrichment analysis; IPS, Immunophenoscore; RF, random forest; ROC, receiver operating characteristic.
