# Supplementary material for: Comprehensive Analysis of Clinical Significance, Immune Infiltration and Biological Role of m6A Regulators in Early-Stage Lung Adenocarcinoma
Source: Front Immunol. 2021 Sep 28;12:698236. doi: 10.3389/fimmu.2021.698236 (PMC8505809; doi:10.3389/fimmu.2021.698236)
Supplement: Supplementary file 1 [file DataSheet_1.pdf]

Supplementary material

Figure S1. The flow chart of our study.

Figure S2. The prognostic value (overall survival) of 23 m^6A regulators in the meta-GEO cohort. (A) Univariate Cox regression analysis of the 23 m^6A regulators. (B) Multivariate Cox regression analysis of the 23 m^6A regulators.

Figure S3. NMF clustering based on 23 m^6A regulators in different cohorts. (A) The results of NMF clustering with different cluster numbers (2, 3, 4, 5, 6) in the meta-GEO cohort. (B) The association between cluster number and cophenetic, residuals, sparseness, dispersion, rss, evar, silhouette coefficients in meta-GEO cohort. (C-D) The heatmap reveals the NMF clustering of 23 m^6A regulators in meta-GEO cohort (C) and TCGA cohort (D). The annotations of patients include GSE group, stage, age, gender, m^6A cluster, Tstage and Nstage.

Figure S4. The overall survival of three m^6A related clusters and the immune landscape of three m^6A gene-related clusters. Multivariate Cox regression analysis revealing the prognostic value (overall survival) of clinical parameters in meta-GEO (A) and TCGA cohorts (B). (B) The Log-rank test was applied to analyze the overall survival (OS) of patients in the meta-GEO cohort among different m^6A related clusters and presented in Kaplan-Meier curves. (C) The Log-rank test was utilized to analyze the overall survival (OS) of patients in the TCGA cohort among **three** m^6A related clusters and presented in Kaplan-Meier curves. (E) The heatmap of the ssGSEA analysis reveals different immune patterns in three m^6A gene-related clusters. The sample annotations include GSE group, stage, age, gender and m^6A gene-related cluster.

Figure S5. The role of LRPPRC and the relationships between 23 m^6A regulators and immune infiltration in meta-GEO cohort. (A) The associations between 23 m^6A regulators and immune infiltrating cells. (B) The fractions of immune infiltrating cells calculated via CIBERSORT algorithm in high- and low-expression group of LRPPRC (*P < 0.05; **P < 0.01; ***P < 0.001). (C) Comparison of immune scores (ImmuneScore, StromalScore, ESTIMATEScore) between high- and low-expression groups of LRPPRC. (D) Comparison of immune scores (PD1, CTLA-4, PD-L1) between high- and low-expression groups of LRPPRC. (E) GSEA enrichment analysis (GO, KEGG) of the top differentially expressed genes in the group with low LRPPRC expression levels.

Figure S6. NMF clustering based on differentially expressed genes among three m^6A clusters in the meta-GEO cohort. (A) The results of NMF clustering with three clusters in the meta-GEO cohort. (B) The association between cluster number and cophenetic, residuals, sparseness, dispersion, rss, evar, silhouette coefficients in the meta-GEO cohort. (C) The heatmap reveals the NMF clustering of differentially expressed genes. The sample annotations include cluster, GSE group, cluster-R, stage, age and gender. (D) Comparison of diverse immune-related scores (ESTIMATEScore, ImmuneScore and StromalScore) in three cluster-Rs (*P < 0.05; **P < 0.01; ***P < 0.001).

Figure S7. The predictive value of the m^6A-predictive score in different cohorts. (A) The heatmap of the key genes and the distribution of the predictive score in meta-GEO, TCGA and GSE72094 cohorts. (B) Time-dependent ROC curves of the m^6A-

predictive score reveal the predictive capability of the m^6A-predictive score in meta-GEO, TCGA and GSE72094 cohorts (AUC: Area under curve).

Figure S8. Establishment of the nomogram in the meta-GEO cohort. (A) Univariate and multivariate Cox regression analyses of the relationship between clinical features and overall survival (OS). (B) The C-index of the nomogram and other clinical parameters. (C) The nomogram used to predict 1-year, 3-year and 5-year early-stage LUAD patients' OS. (D) Time-dependent ROC curves utilized to evaluate the 1-year, 3-year and 5-year predictive capability of the nomogram (AUC: Area under curves). (E) Calibration curves used to evaluate the nomogram's predictive efficiency.

Figure S9. The m^6A genes in T cells of LUAD by single-cell sequencing. (A) WTAP expression in various T cells populations. (B) WTAP expression in individual T cells. The color represents the expression levels and each dot represents a single cell. (C) CBLL1 expression in various T cells populations. (D) WTAP expression in individual T cells. (E) The t-SNE plot indicating the distribution of different T cells clusters.

Table S1. The classification of 23 m^6A regulators.

Table S2. Basic information of early-stage LUAD samples in this study.

Table S3. Correlation of m^6A regulators and DNA methylation in the TCGA cohort.

Table S4. Correlation among expression levels of m^6A regulators.

Table S5. Information of m^6A clusters, m^6A cluster-Rs and m^6A-score groups in the meta-GEO cohort.

Table S6. Information of m^6A clusters and m^6A-score groups in the TCGA cohort.

Table S7. Univariate Cox regression analysis of the differentially expressed genes among three clusters.

Table S8. Multivariate Cox regression analysis of the genes selected by the Random Forest algorithm.
