# Supplementary material for: Comprehensive Analysis of Clinical Significance, Immune Infiltration and Biological Role of m6A Regulators in Early-Stage Lung Adenocarcinoma
Source: Front Immunol. 2021 Sep 28;12:698236. doi: 10.3389/fimmu.2021.698236 (PMC8505809; doi:10.3389/fimmu.2021.698236)
Supplement: Supplementary file 2 [file Image_1.pdf]

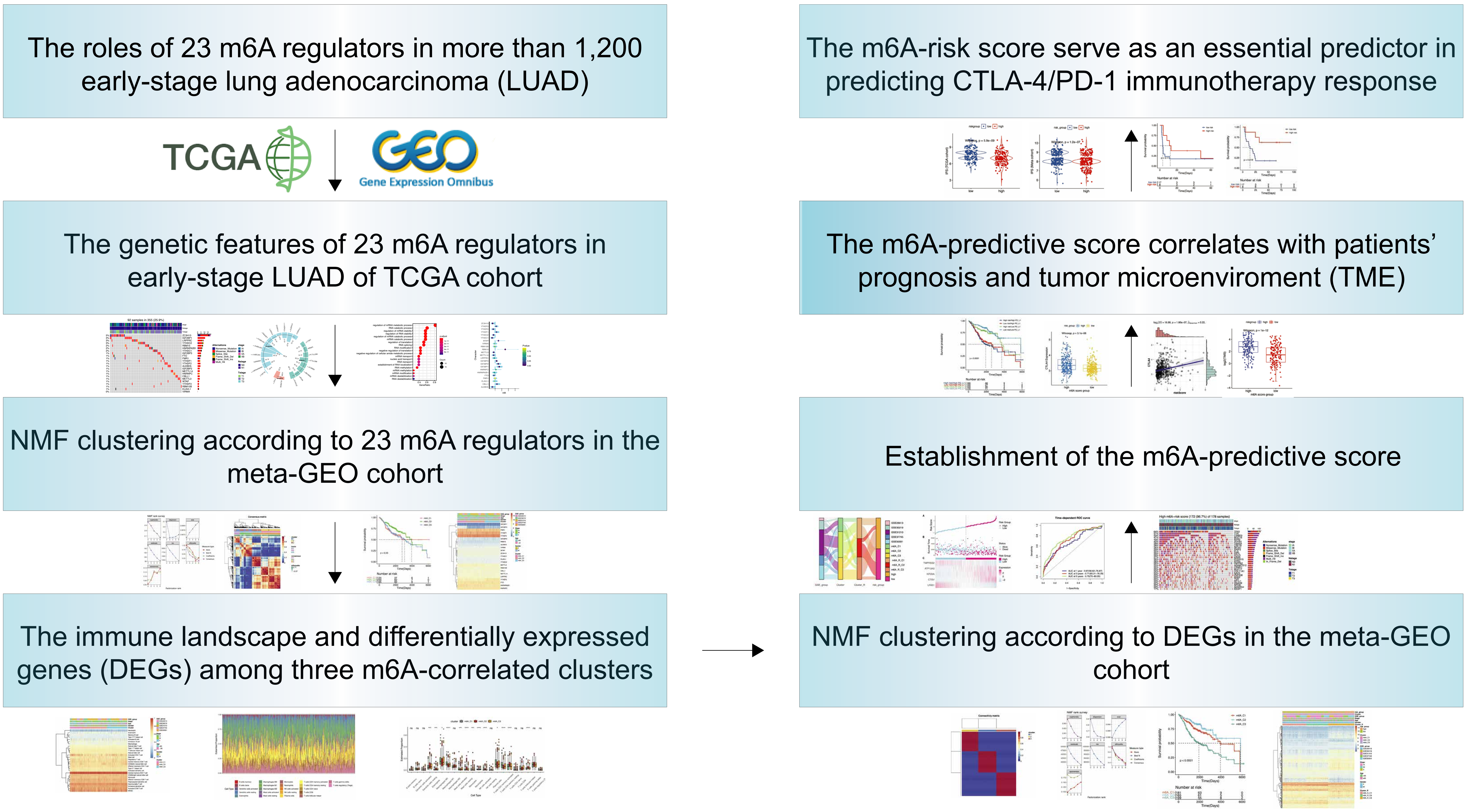
The roles of 23 m6A regulators in more than 1,200 early-stage lung adenocarcinoma (LUAD)
TCGA
GEO
Gene Expression Omnibus
The genetic features of 23 m6A regulators in early-stage LUAD of TCGA cohort
NMF clustering according to 23 m6A regulators in the meta-GEO cohort
The immune landscape and differentially expressed genes (DEGs) among three m6A-correlated clusters
NMF clustering according to DEGs in the meta-GEO cohort
Establishment of the m6A-predictive score
The m6A-predictive score correlates with patients' prognosis and tumor microenviroment (TME)
The m6A-risk score serve as an essential predictor in predicting CTLA-4/PD-1 immunotherapy response
