# Supplementary figures and images for: Comprehensive Analysis of Clinical Significance, Immune Infiltration and Biological Role of m6A Regulators in Early-Stage Lung Adenocarcinoma
Source: Front Immunol. 2021 Sep 28;12:698236. doi: 10.3389/fimmu.2021.698236 (PMC8505809; doi:10.3389/fimmu.2021.698236)

**A****Univariate Cox Regression**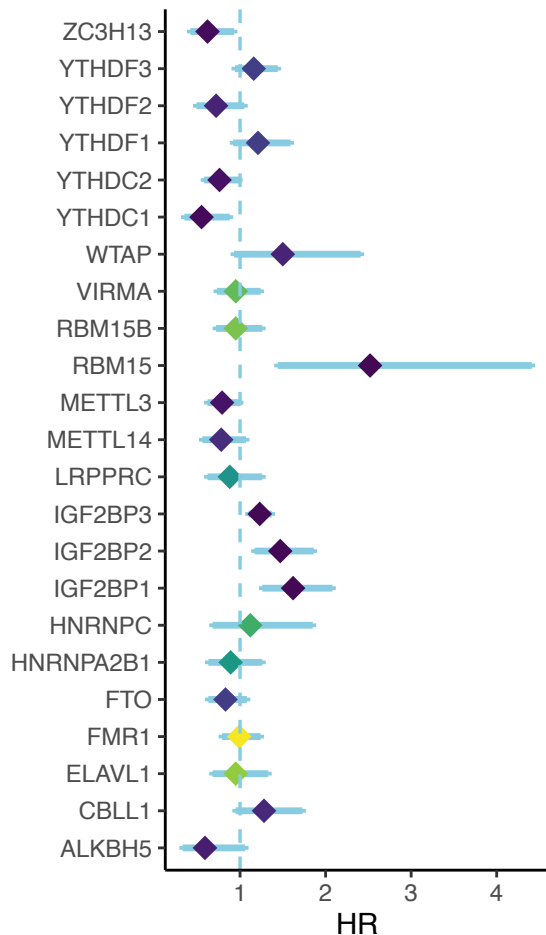**B****Multivariate Cox Regression**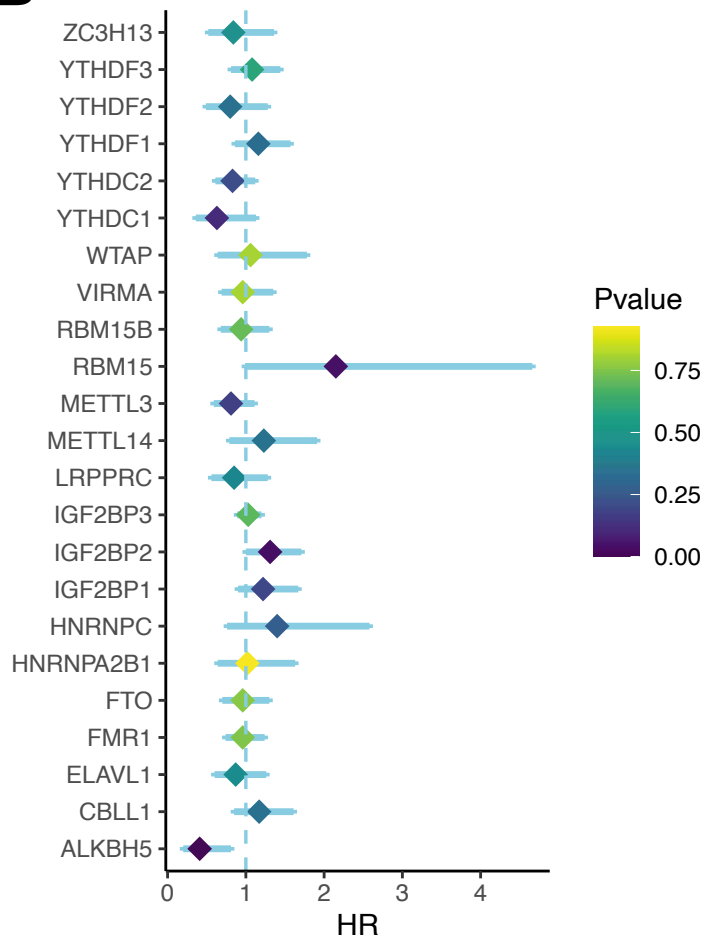

Supplement: Supplementary file 3 [file Image_2.pdf]

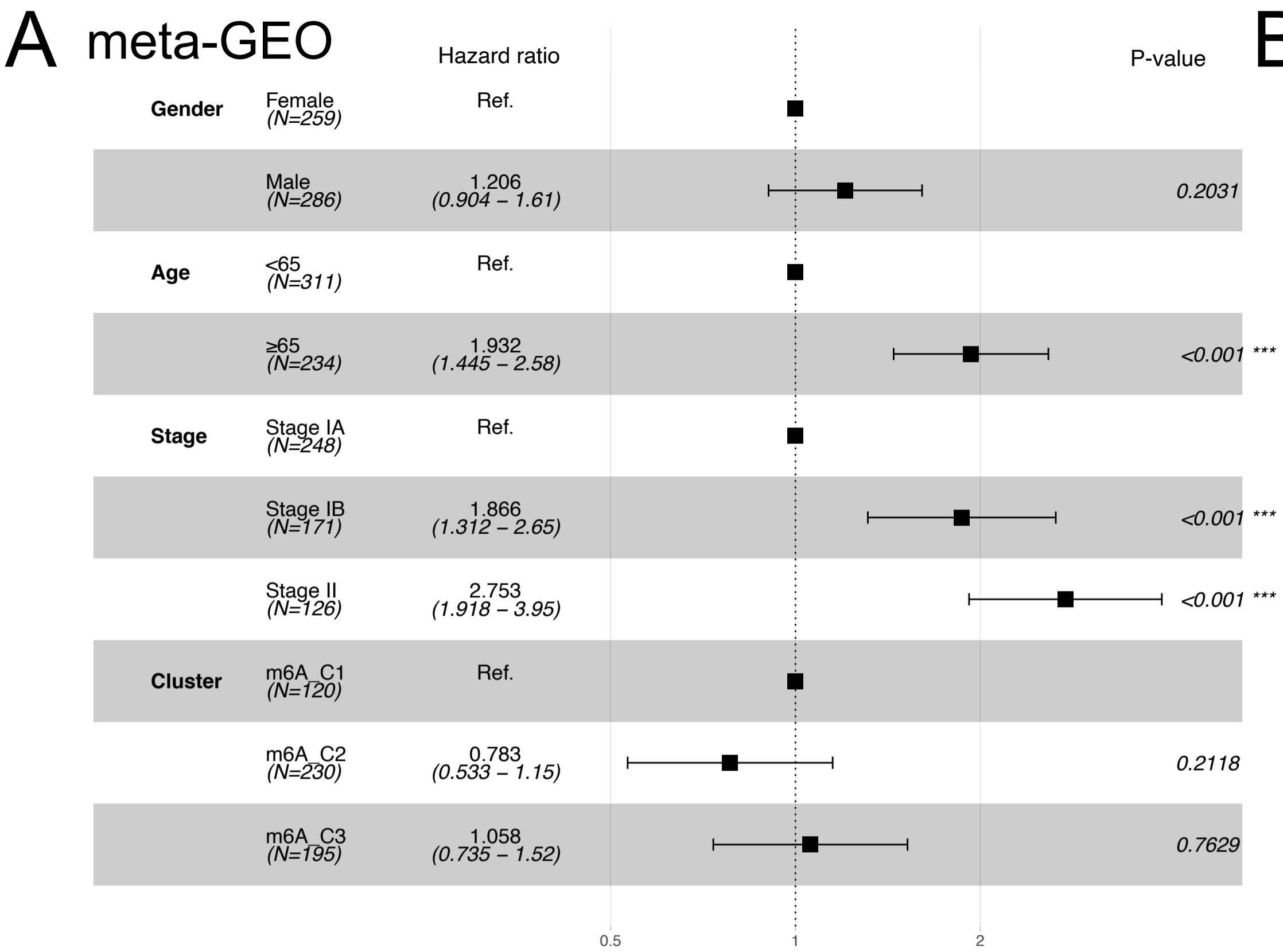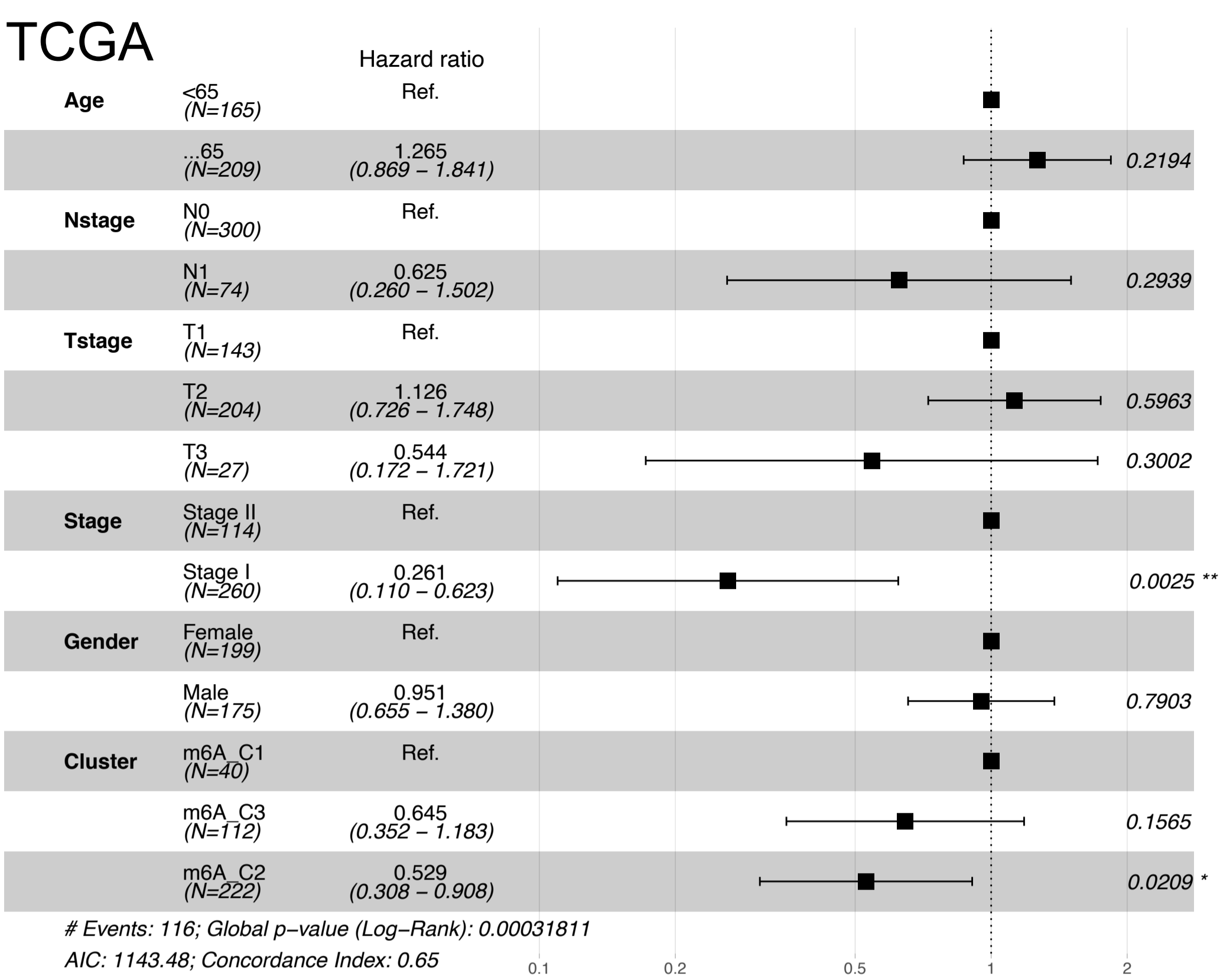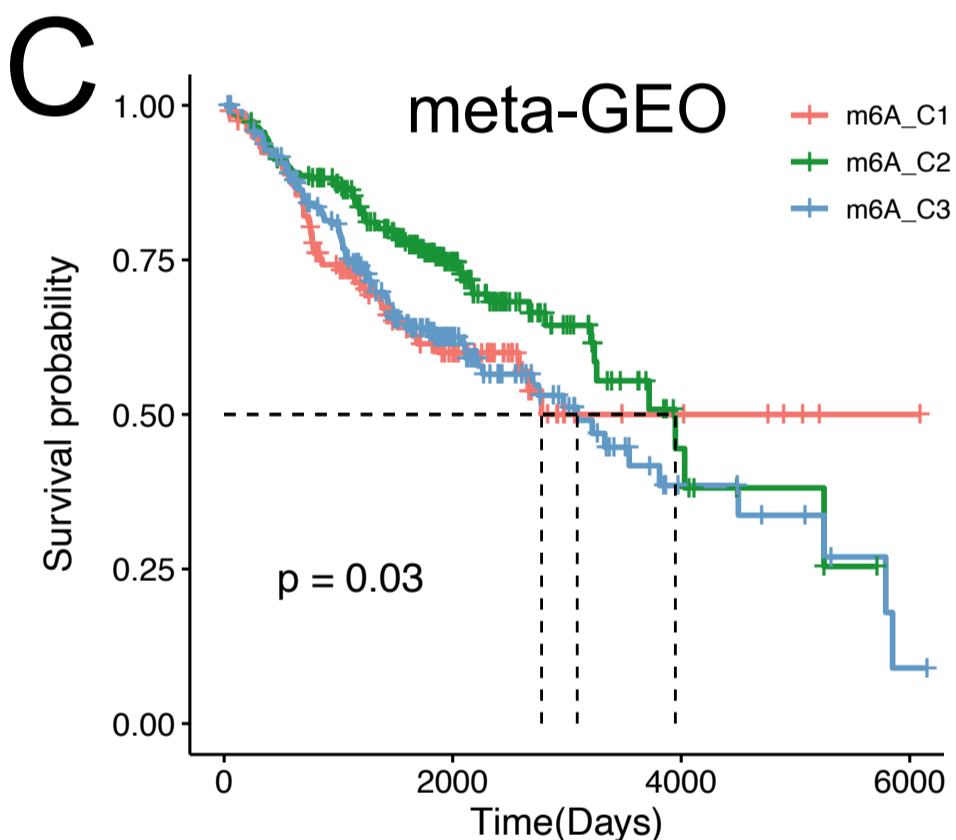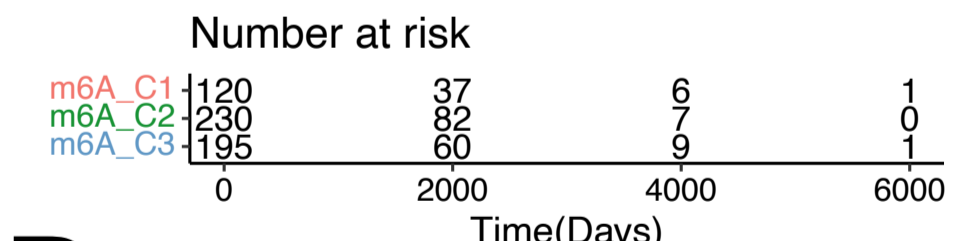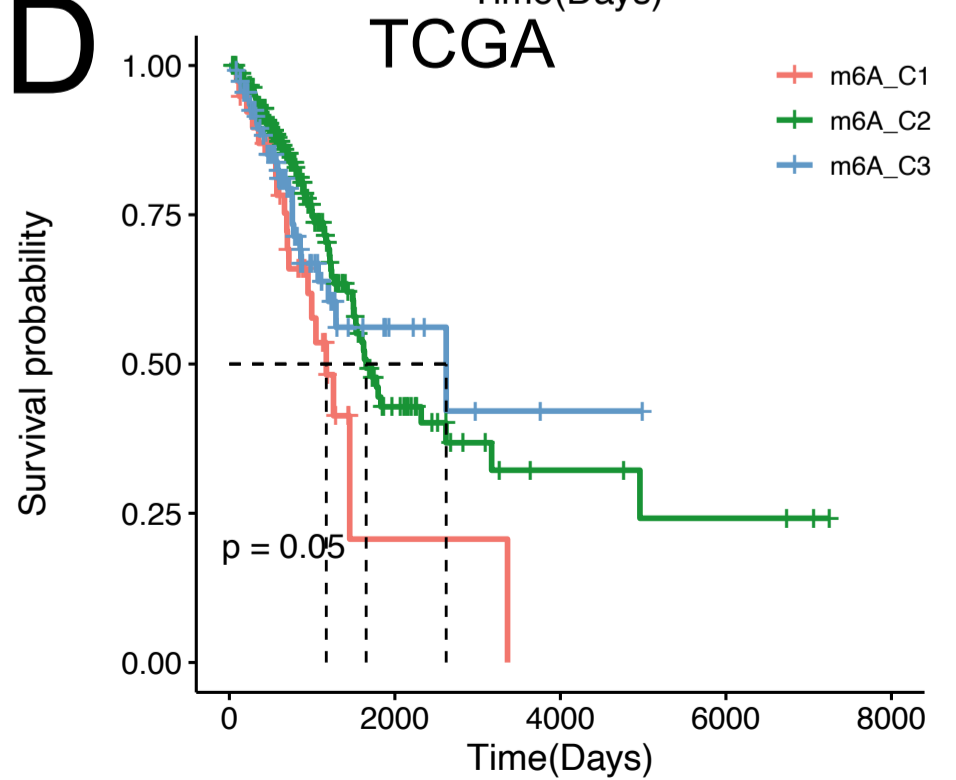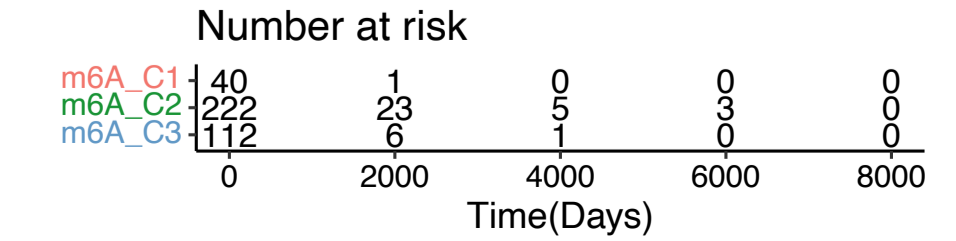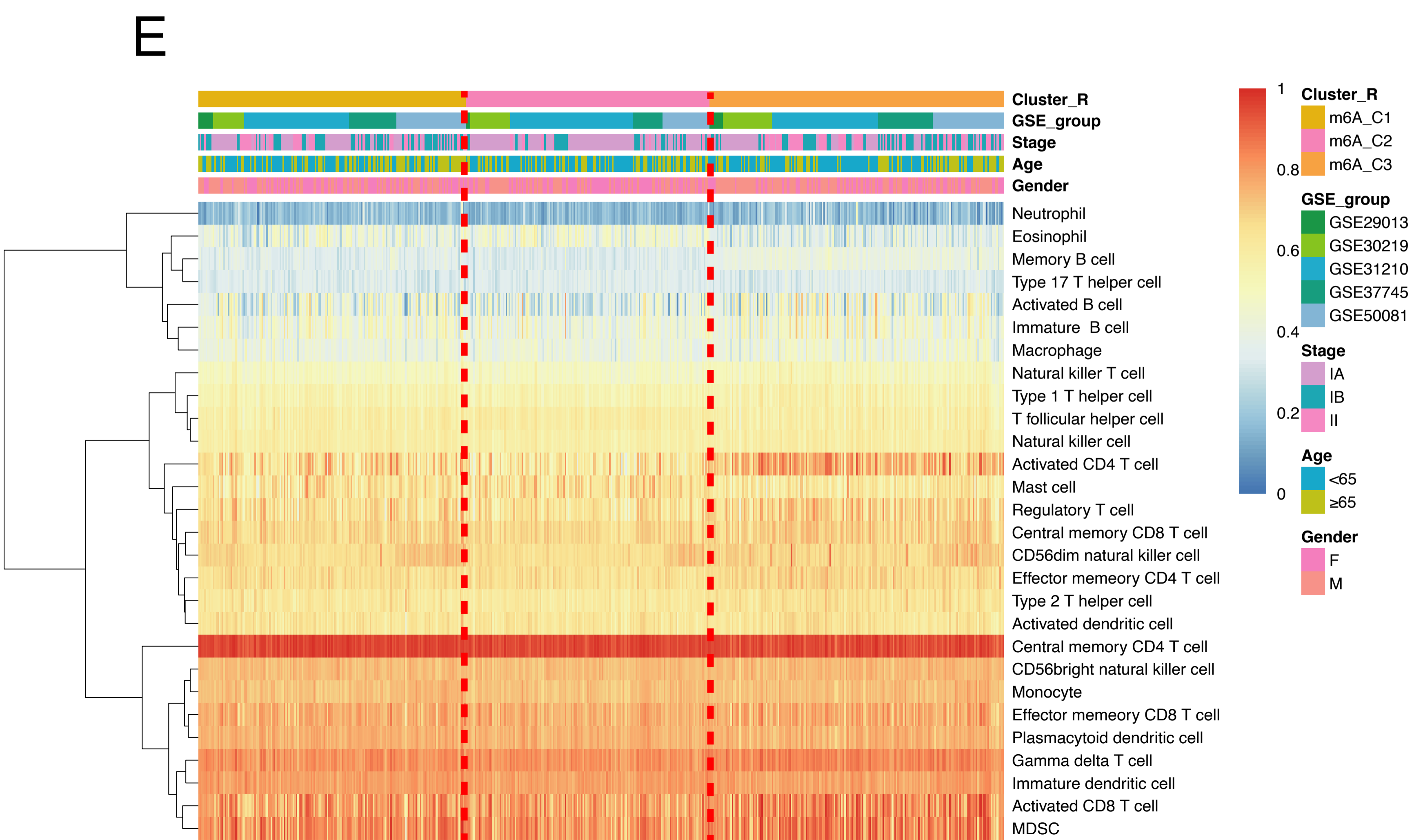

Supplement: Supplementary file 5 [file Image_4.pdf]

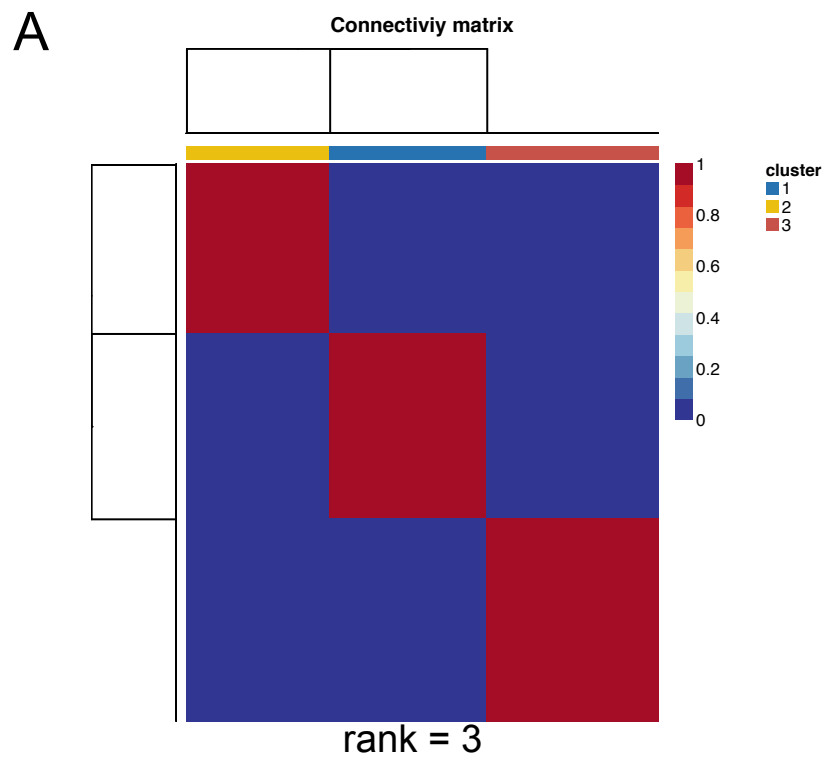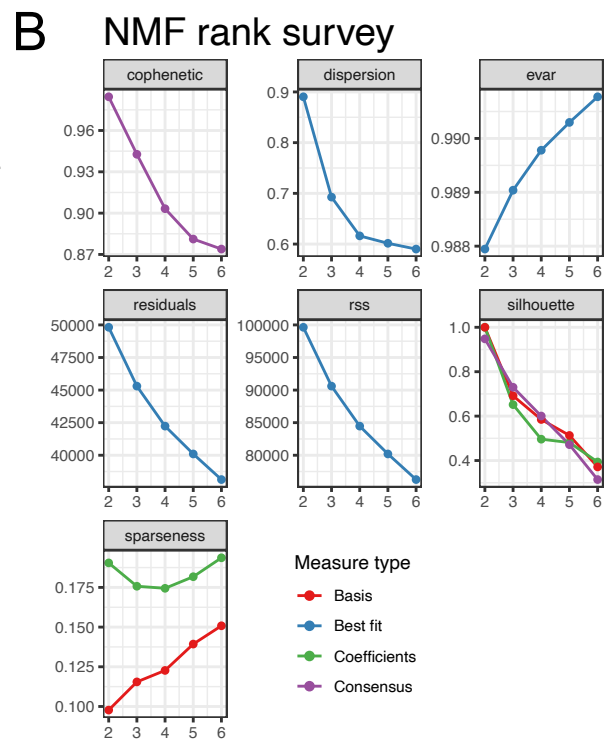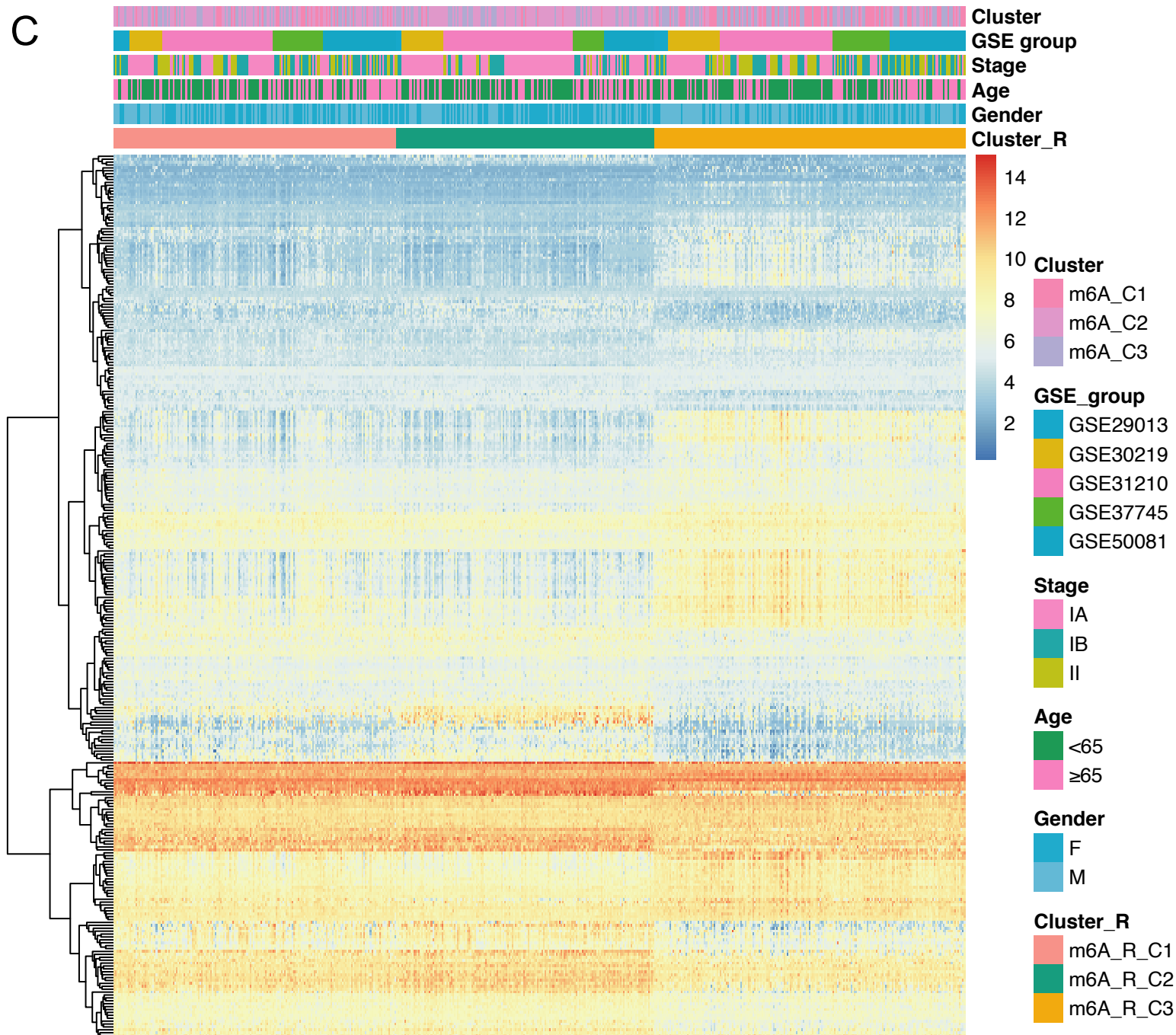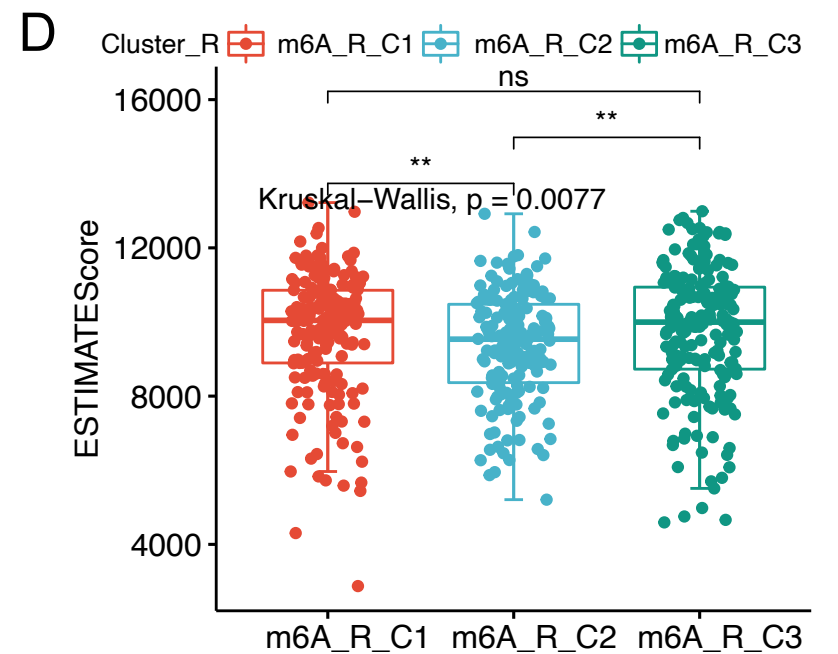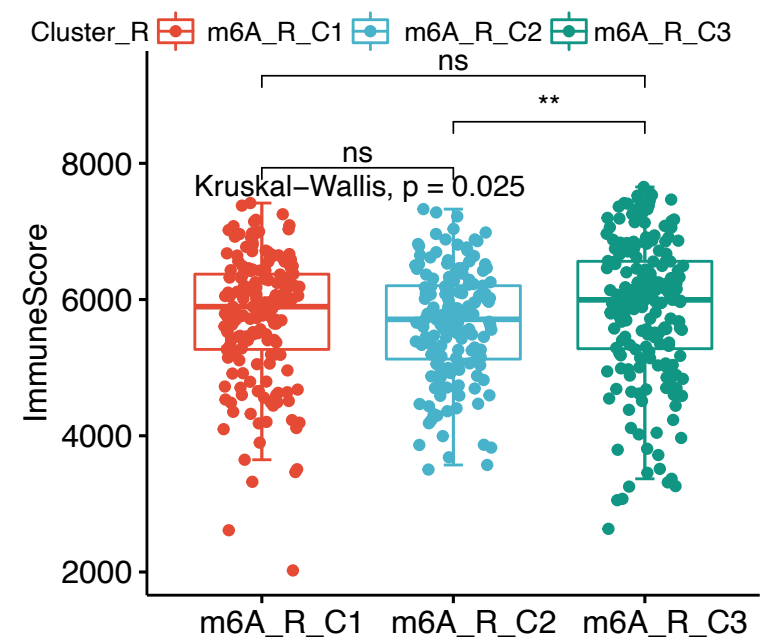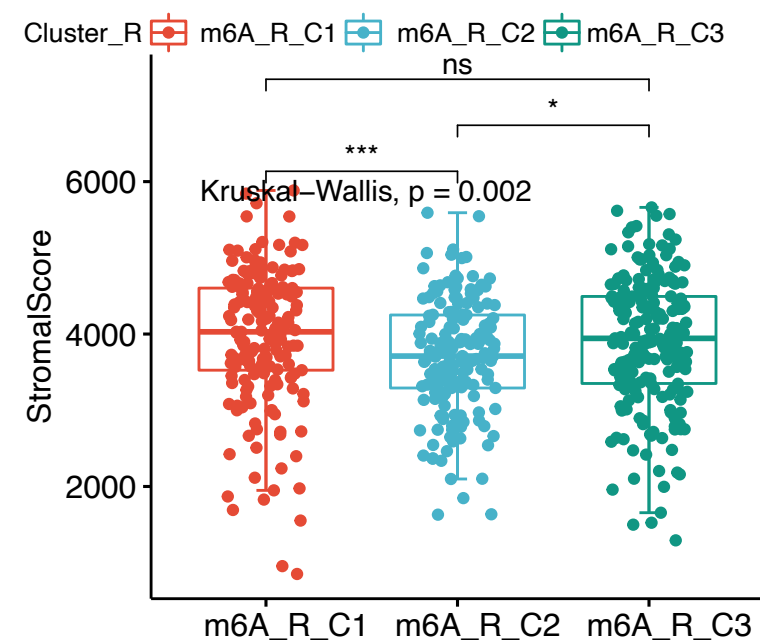

Supplement: Supplementary file 7 [file Image_6.pdf]

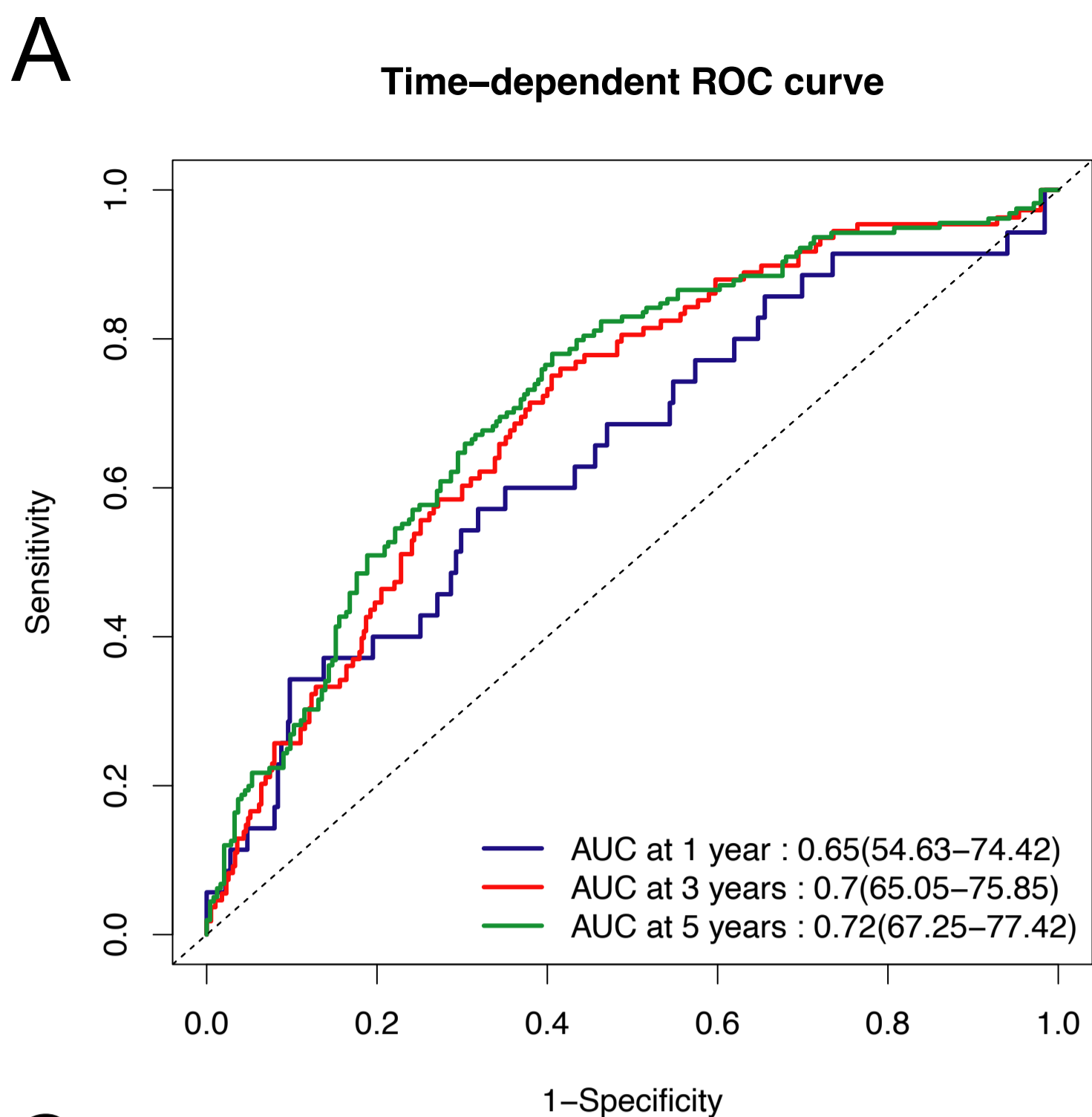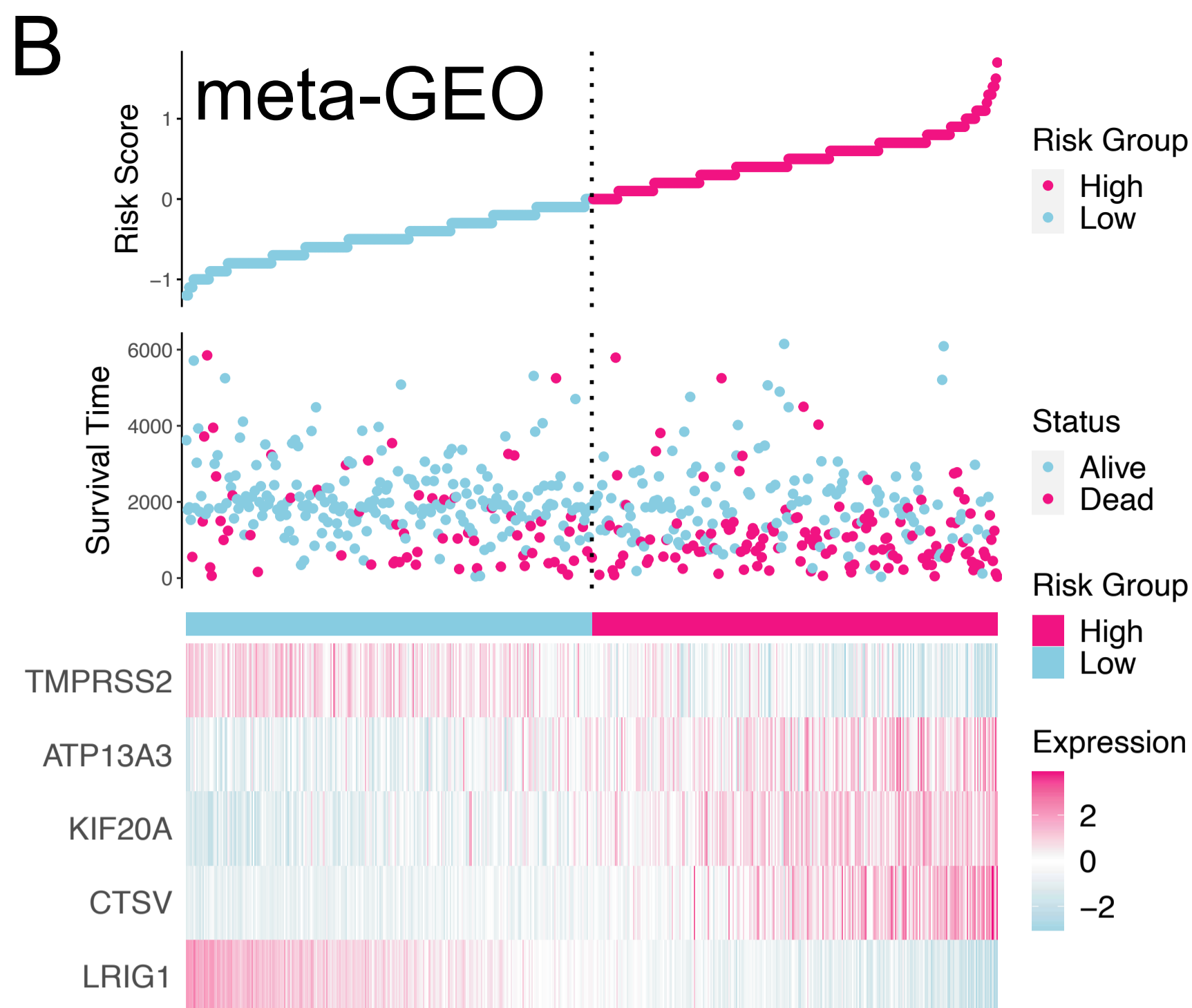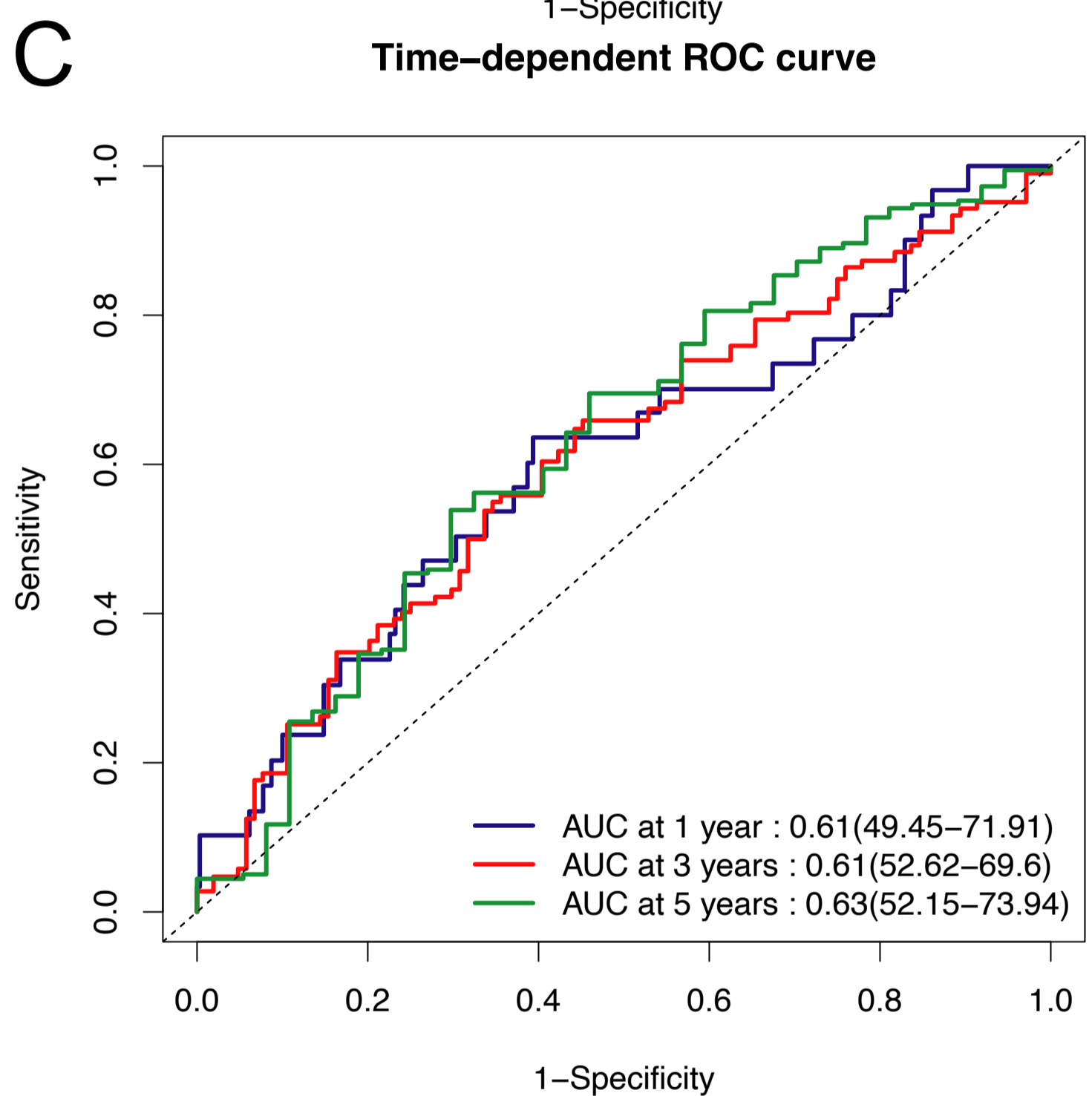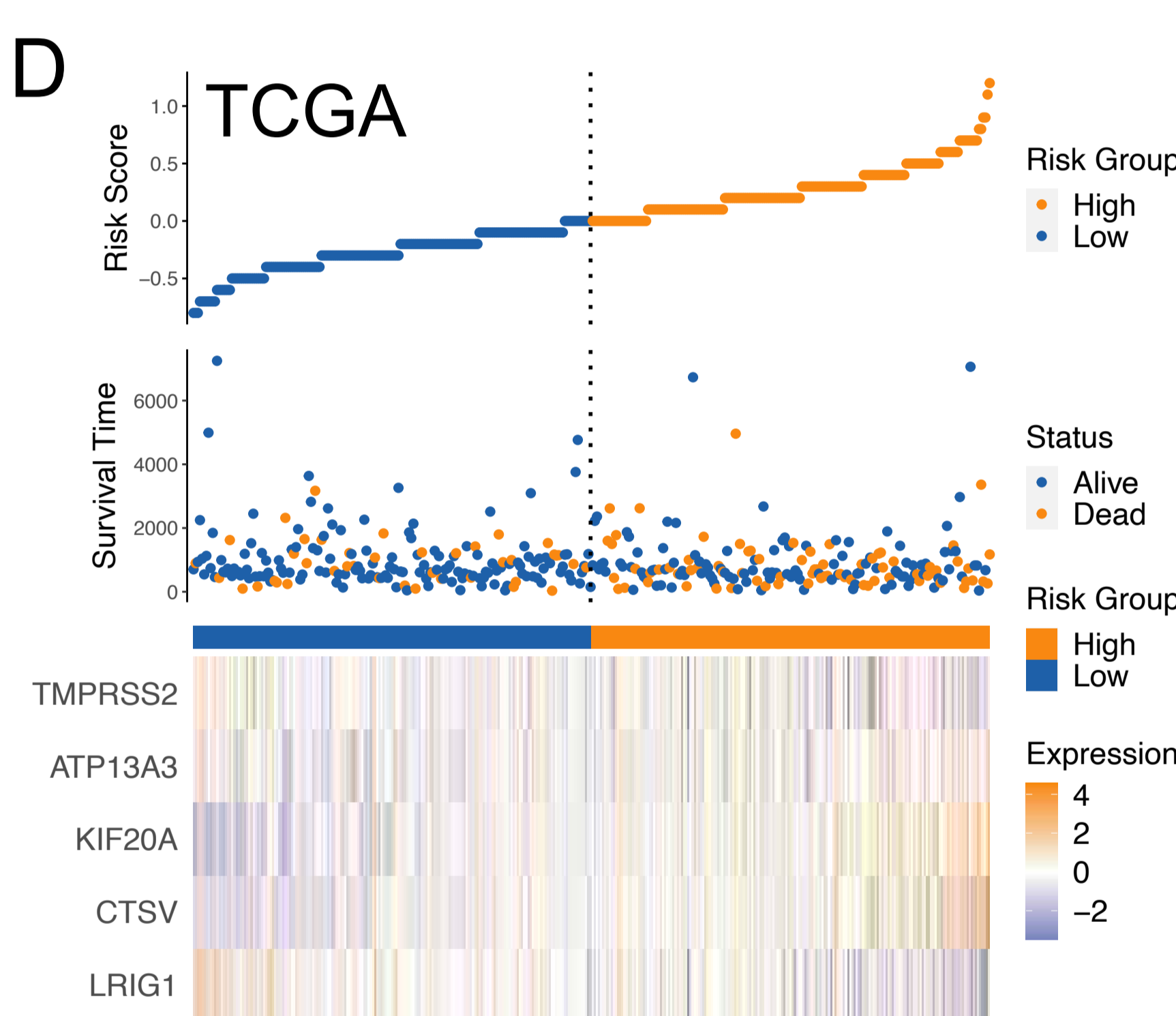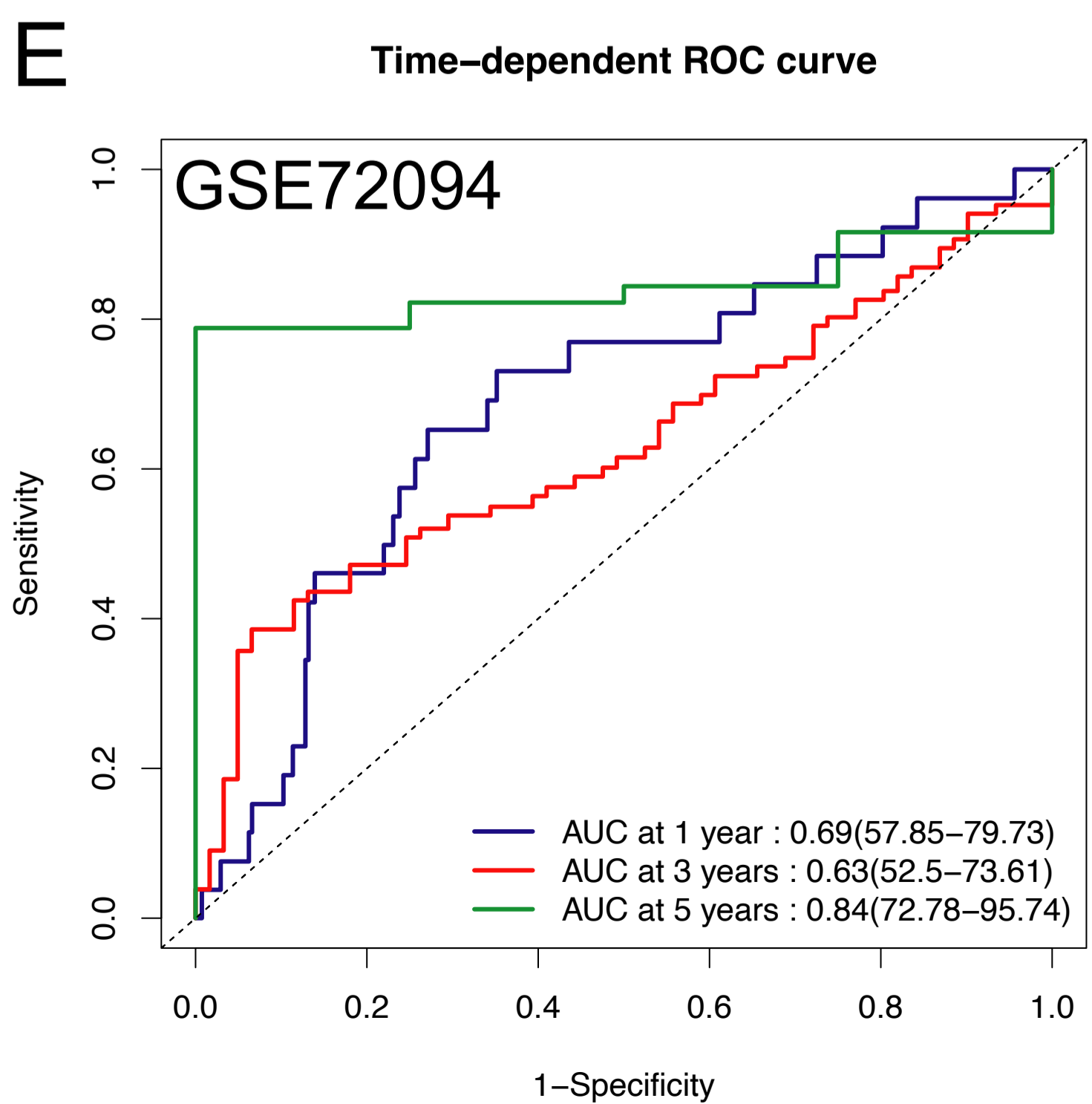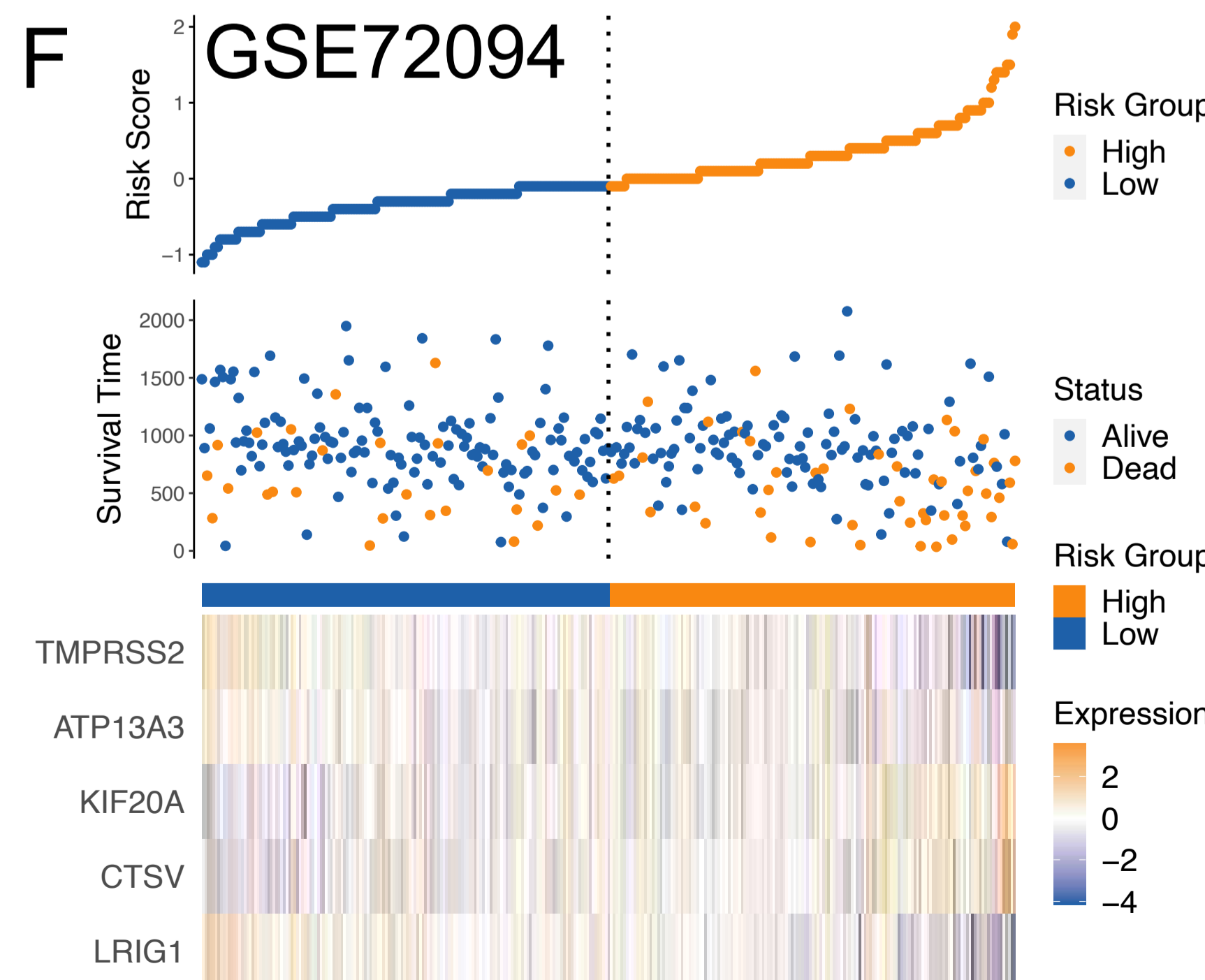

Supplement: Supplementary file 8 [file Image_7.pdf]

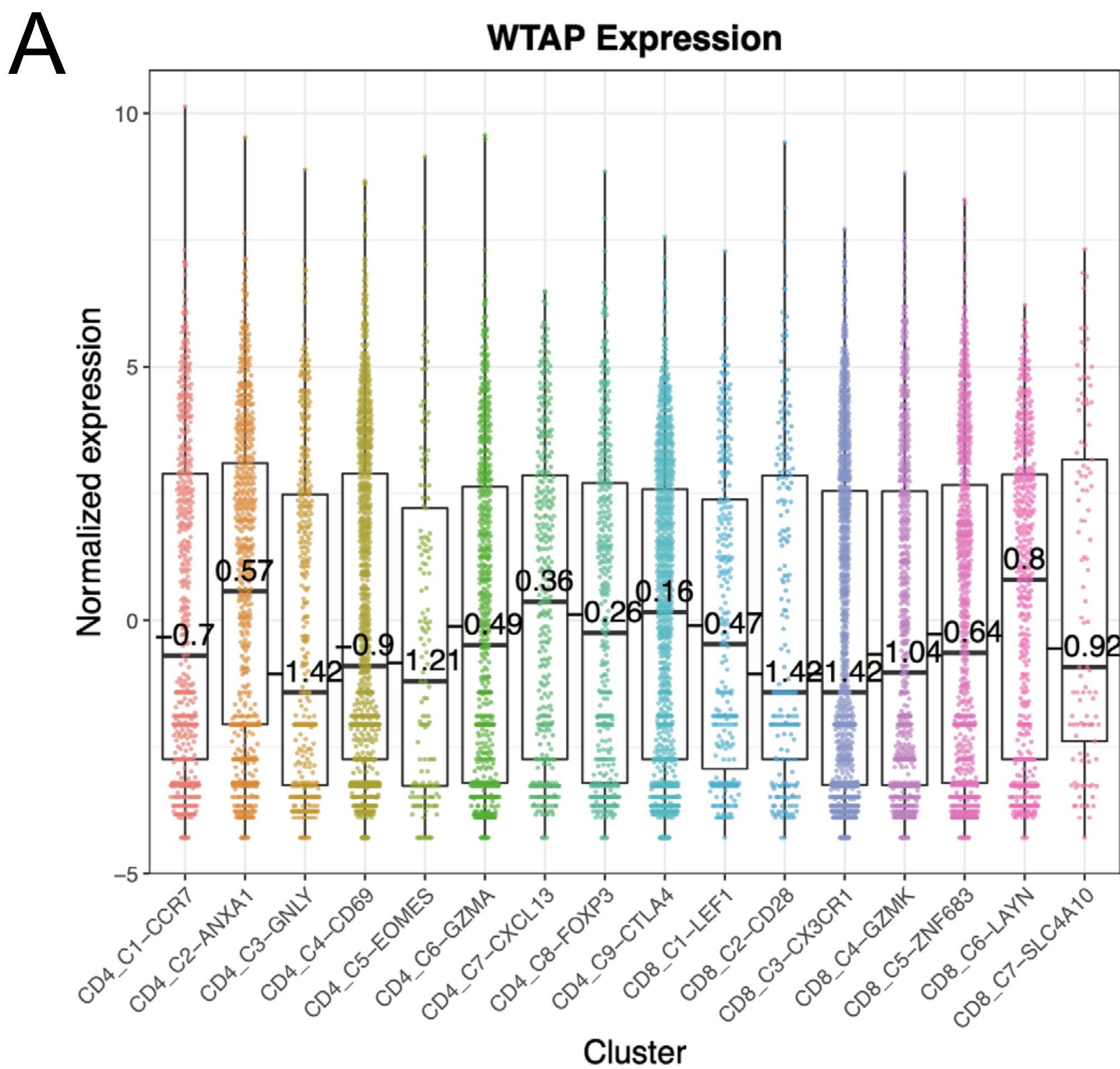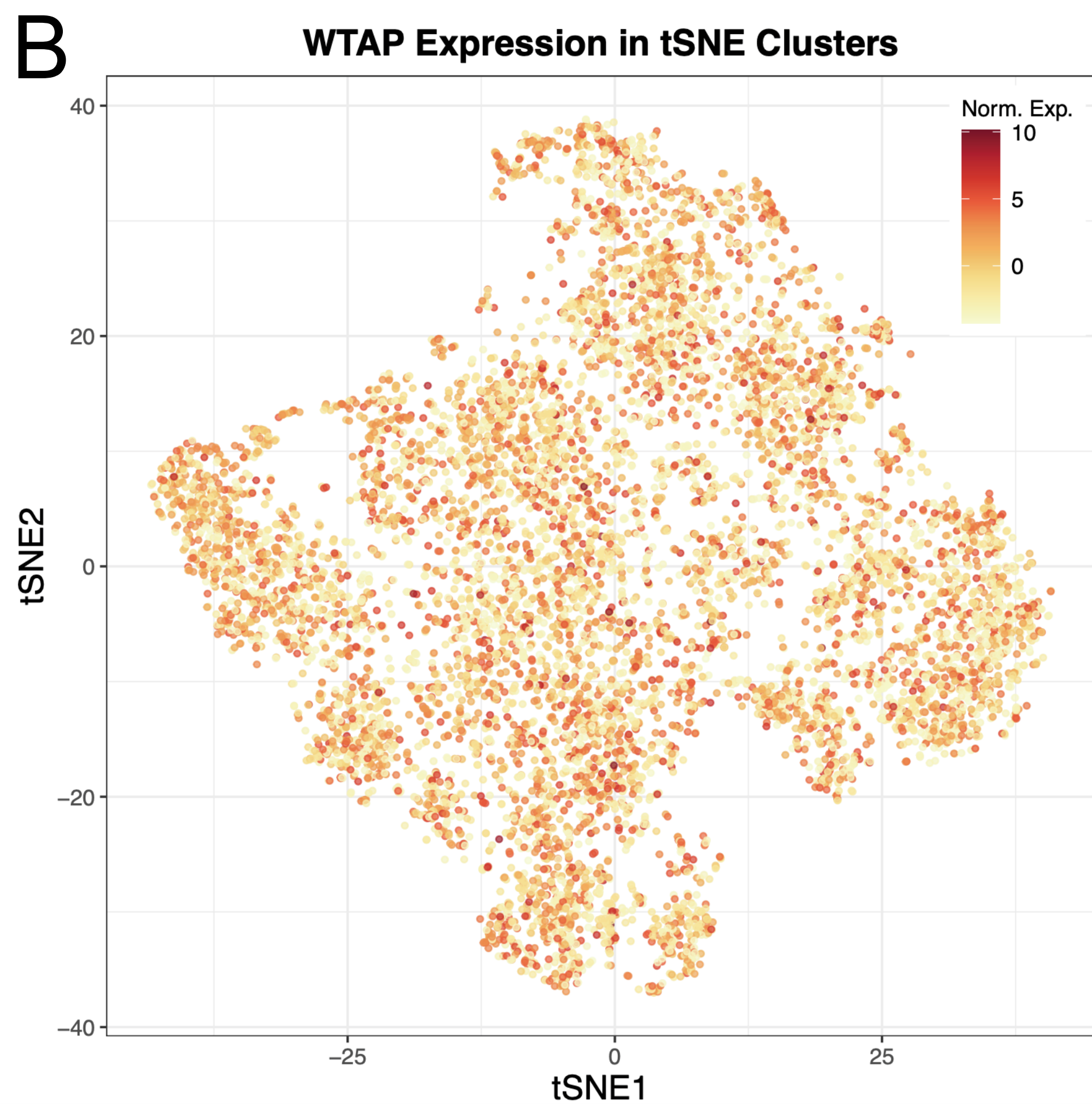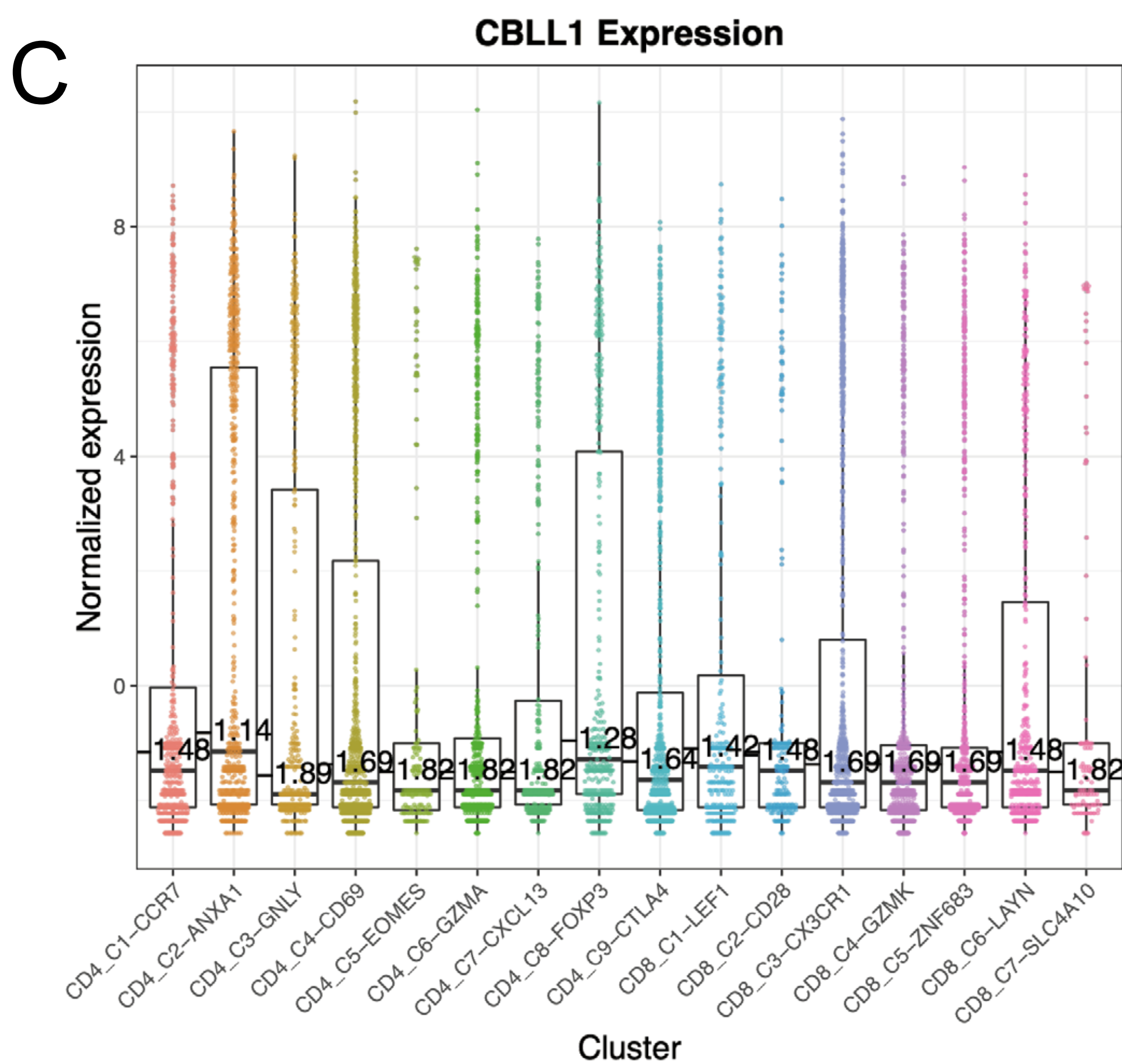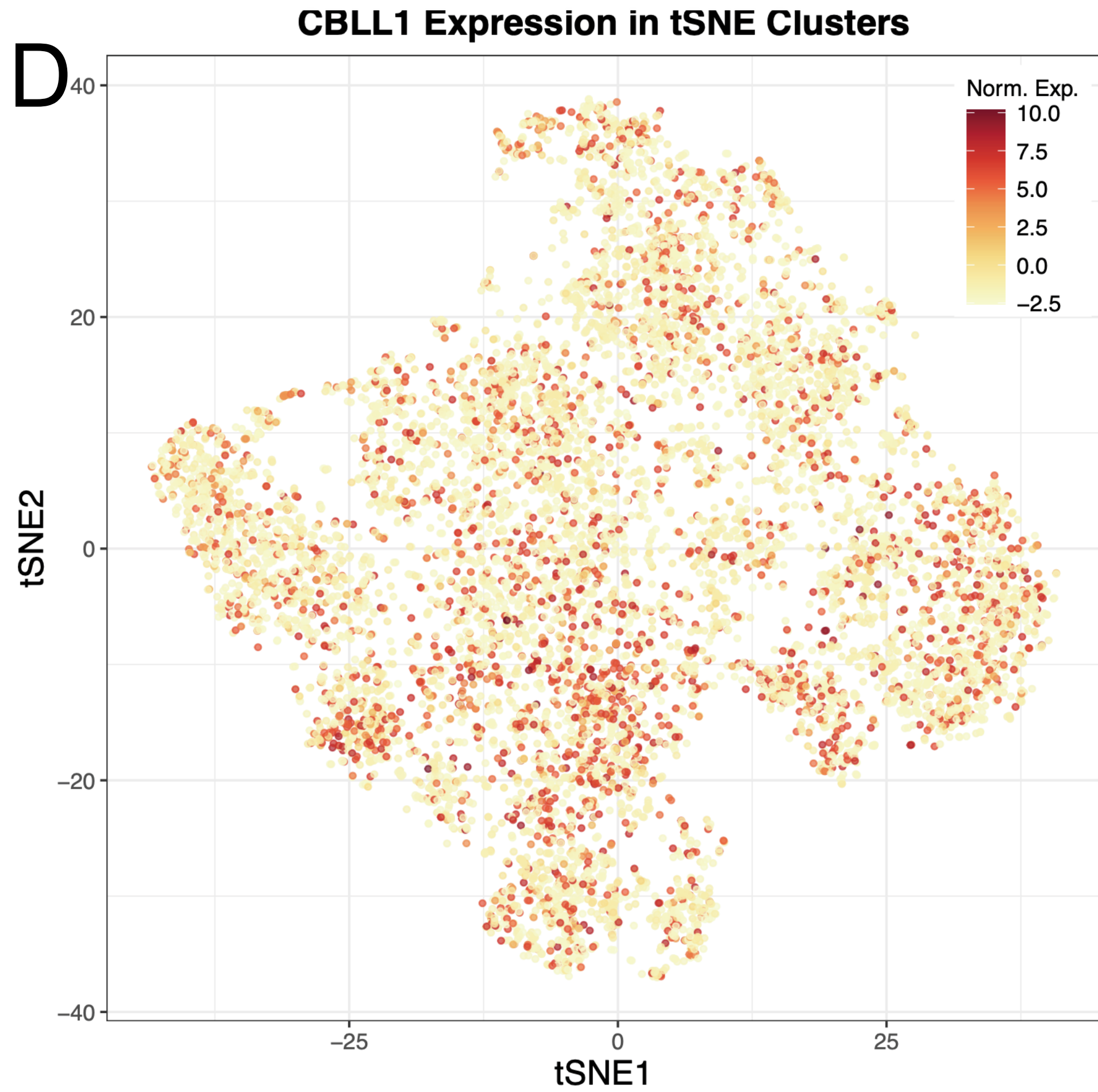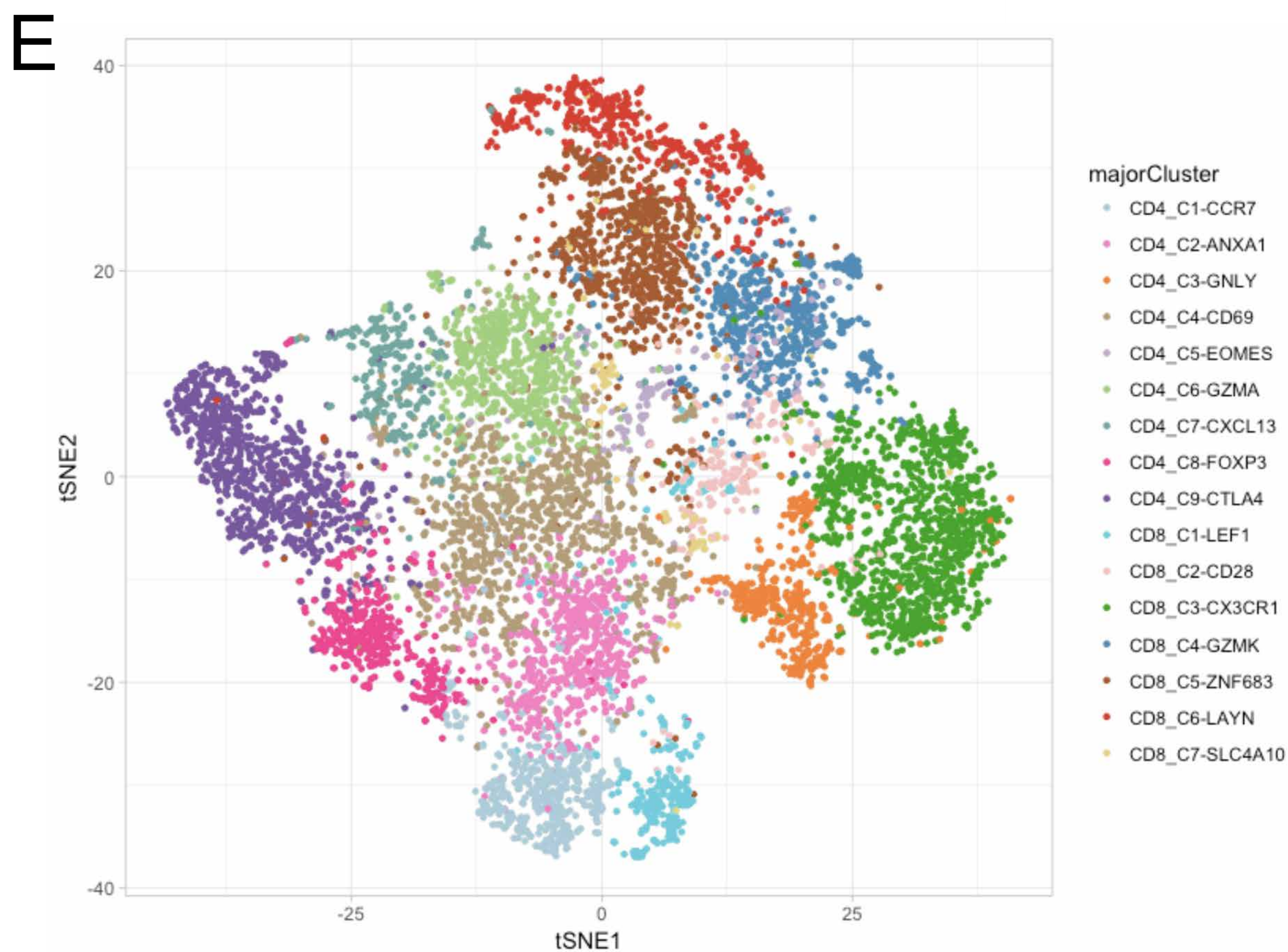

Supplement: Supplementary file 10 [file Image_9.pdf]
