# Supplementary material for: Comprehensive Analysis of Clinical Significance, Immune Infiltration and Biological Role of m6A Regulators in Early-Stage Lung Adenocarcinoma
Source: Front Immunol. 2021 Sep 28;12:698236. doi: 10.3389/fimmu.2021.698236 (PMC8505809; doi:10.3389/fimmu.2021.698236)
Supplement: Supplementary file 4 [file Image_3.pdf]

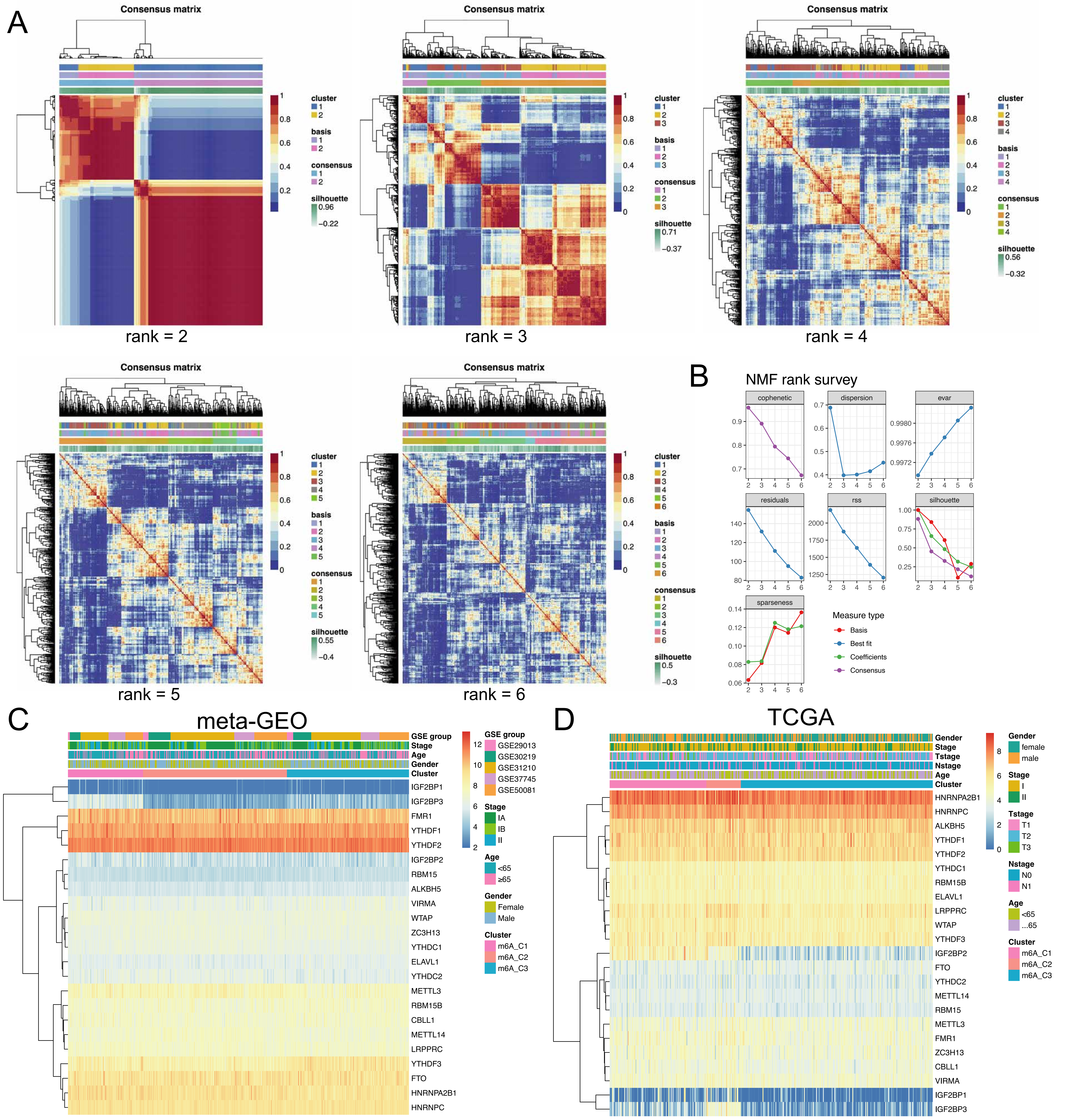

A
Consensus matrix
rank = 2
rank = 3
rank = 4
rank = 5
rank = 6
cluster
basis
consensus
silhouette
B
NMF rank survey
cophenetic
dispersion
evar
residuals
rss
silhouette
sparseness
Measure type
Basis
Best fit
Coefficients
Consensus
C
meta-GEO
GSE group
Stage
Age
Gender
Cluster
GSE29013
GSE30219
GSE31210
GSE37745
GSE50081
IA
IB
II
<65
≥65
Female
Male
m6A_C1
m6A_C2
m6A_C3
IGF2BP1
IGF2BP3
FMR1
YTHDF1
YTHDF2
IGF2BP2
RBM15
ALKBH5
VIRMA
WTAP
ZC3H13
YTHDC1
ELAVL1
YTHDC2
METTL3
RBM15B
CBLL1
METTL14
LRPPRC
YTHDF3
FTO
HNRNPA2B1
HNRNPC
D
TCGA
Gender
Stage
Tstage
Nstage
Age
Cluster
female
male
I
II
T1
T2
T3
N0
N1
<65
...65
m6A_C1
m6A_C2
m6A_C3
HNRNPA2B1
HNRNPC
ALKBH5
YTHDF1
YTHDF2
YTHDC1
RBM15B
ELAVL1
LRPPRC
WTAP
YTHDF3
IGF2BP2
FTO
YTHDC2
METTL14
RBM15
METTL3
FMR1
ZC3H13
CBLL1
VIRMA
IGF2BP1
IGF2BP3
