# Supplementary material for: Comprehensive Analysis of Clinical Significance, Immune Infiltration and Biological Role of m6A Regulators in Early-Stage Lung Adenocarcinoma
Source: Front Immunol. 2021 Sep 28;12:698236. doi: 10.3389/fimmu.2021.698236 (PMC8505809; doi:10.3389/fimmu.2021.698236)
Supplement: Supplementary file 6 [file Image_5.pdf]

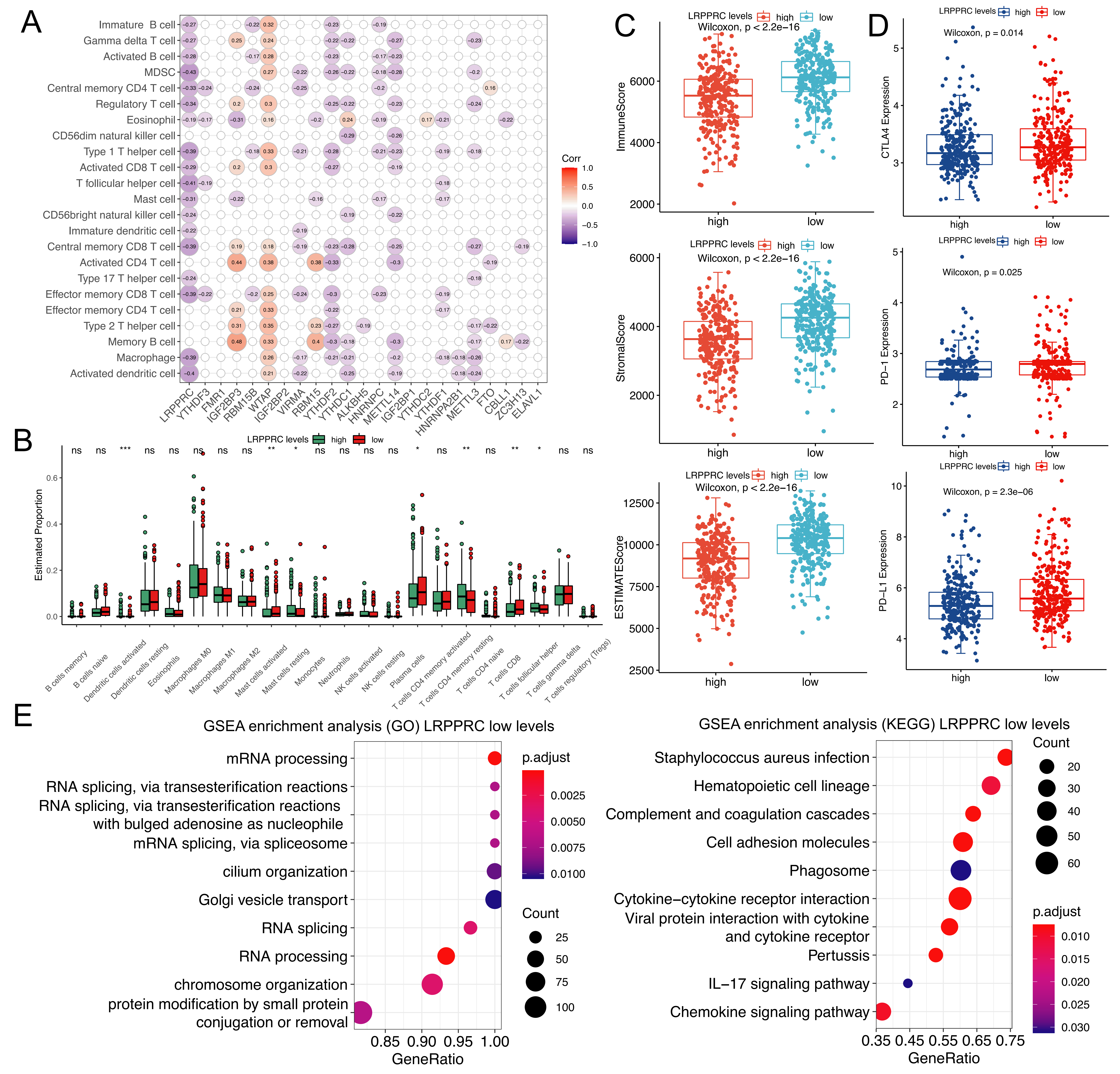

A
Immature B cell
Gamma delta T cell
Activated B cell
MDSC
Central memory CD4 T cell
Regulatory T cell
Eosinophil
CD56dim natural killer cell
Type 1 T helper cell
Activated CD8 T cell
T follicular helper cell
Mast cell
CD56bright natural killer cell
Immature dendritic cell
Central memory CD8 T cell
Activated CD4 T cell
Type 17 T helper cell
Effector memory CD8 T cell
Effector memory CD4 T cell
Type 2 T helper cell
Memory B cell
Macrophage
Activated dendritic cell
LRPPRC YTHDF3 FMR1 IGF2BP3 RBM15B WTAP IGF2BP2 VIRMA RBM15 YTHDF2 YTHDC1 ALKBH5 HNRNPC METTL14 IGF2BP1 YTHDC2 YTHDF1 HNRNPA2B1 METTL3 FTO CBLL1 ZC3H13 ELAVL1
Corr
1.0 0.5 0.0 −0.5 −1.0
B
LRPPRC levels high low
Estimated Proportion
ns ns *** ns ns ns ns ns ** * ns ns ns ns * ns ** ns ** * ns ns
B cells memory
B cells naive
Dendritic cells activated
Dendritic cells resting
Eosinophils
Macrophages M0
Macrophages M1
Macrophages M2
Mast cells activated
Mast cells resting
Monocytes
Neutrophils
NK cells activated
NK cells resting
Plasma cells
T cells CD4 memory activated
T cells CD4 memory resting
T cells CD4 naive
T cells CD8
T cells follicular helper
T cells gamma delta
T cells regulatory (Tregs)
C
LRPPRC levels high low
Wilcoxon, p < 2.2e−16
ImmuneScore
StromalScore
ESTIMATEScore
high low
D
LRPPRC levels high low
Wilcoxon, p = 0.014
CTLA4 Expression
Wilcoxon, p = 0.025
PD−1 Expression
Wilcoxon, p = 2.3e−06
PD−L1 Expression
E
GSEA enrichment analysis (GO) LRPPRC low levels
mRNA processing
RNA splicing, via transesterification reactions
RNA splicing, via transesterification reactions with bulged adenosine as nucleophile
mRNA splicing, via spliceosome
cilium organization
Golgi vesicle transport
RNA splicing
RNA processing
chromosome organization
protein modification by small protein conjugation or removal
GeneRatio
p.adjust
Count
GSEA enrichment analysis (KEGG) LRPPRC low levels
Staphylococcus aureus infection
Hematopoietic cell lineage
Complement and coagulation cascades
Cell adhesion molecules
Phagosome
Cytokine−cytokine receptor interaction
Viral protein interaction with cytokine and cytokine receptor
Pertussis
IL−17 signaling pathway
Chemokine signaling pathway
GeneRatio
Count
p.adjust
