# Supplementary material for: Comprehensive Analysis of Clinical Significance, Immune Infiltration and Biological Role of m6A Regulators in Early-Stage Lung Adenocarcinoma
Source: Front Immunol. 2021 Sep 28;12:698236. doi: 10.3389/fimmu.2021.698236 (PMC8505809; doi:10.3389/fimmu.2021.698236)
Supplement: Supplementary file 9 [file Image_8.pdf]

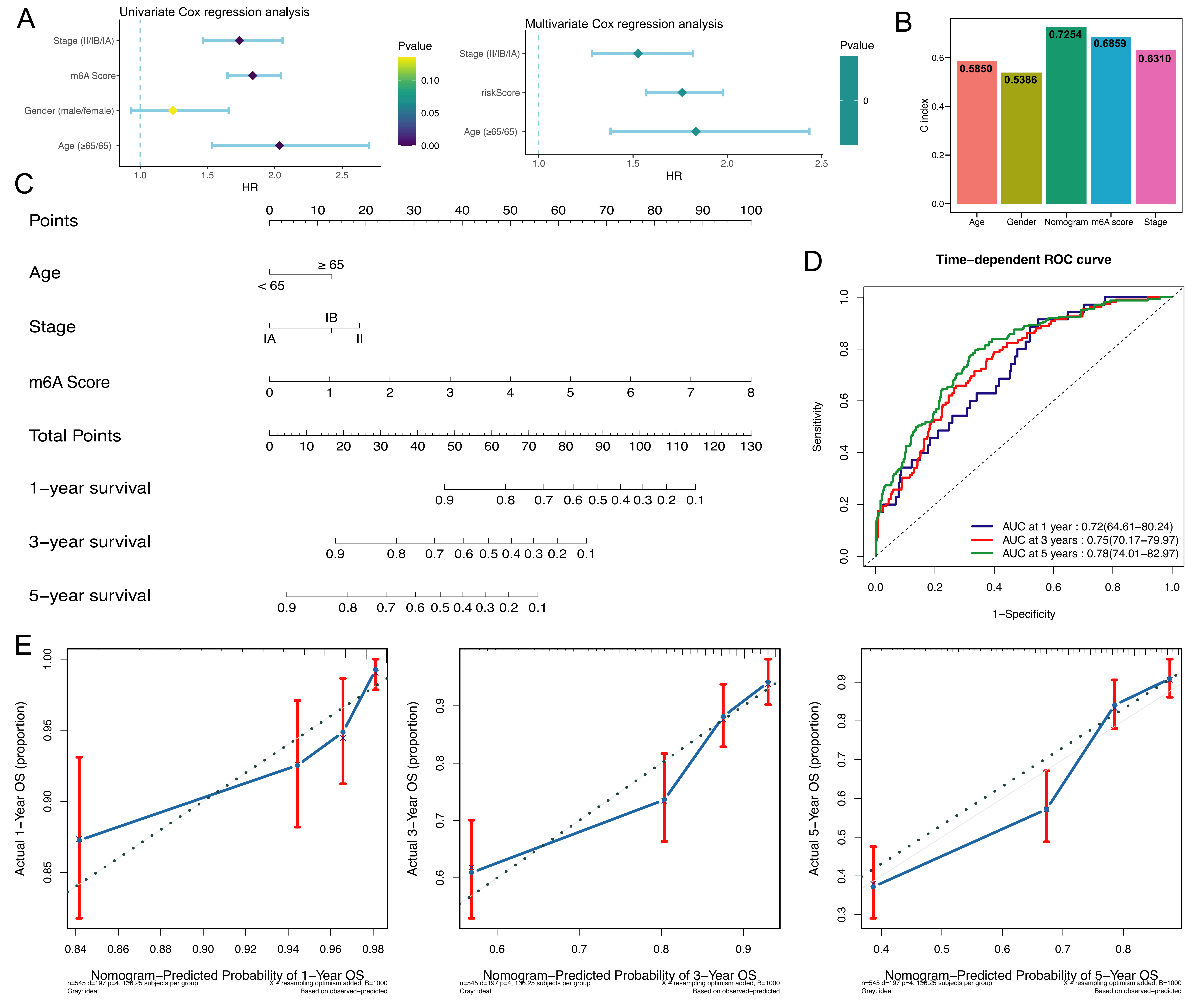

A
Univariate Cox regression analysis
Stage (II/IB/IA)
m6A Score
Gender (male/female)
Age (≥65/65)
HR
Pvalue
Multivariate Cox regression analysis
riskScore
B
C index
0.5850
0.5386
0.7254
0.6859
0.6310
Age
Gender
Nomogram
m6A score
Stage
C
Points
Age
≥ 65
< 65
Stage
IA
IB
II
m6A Score
Total Points
1−year survival
3−year survival
5−year survival
D
Time-dependent ROC curve
Sensitivity
1−Specificity
AUC at 1 year : 0.72(64.61−80.24)
AUC at 3 years : 0.75(70.17−79.97)
AUC at 5 years : 0.78(74.01−82.97)
E
Actual 1-Year OS (proportion)
Nomogram−Predicted Probability of 1−Year OS
Actual 3-Year OS (proportion)
Nomogram−Predicted Probability of 3−Year OS
Actual 5-Year OS (proportion)
Nomogram−Predicted Probability of 5−Year OS
n=545 d=197 p=4, 136.25 subjects per group
Gray: ideal
X − resampling optimism added, B=1000
Based on observed−predicted
